# Experience-based health state valuation using the EQ VAS: a register-based study of the EQ-5D-3L among nine patient groups in Sweden

**DOI:** 10.1186/s12955-023-02115-z

**Published:** 2023-04-10

**Authors:** Fitsum Sebsibe Teni, Kristina Burström, Nancy Devlin, David Parkin, Ola Rolfson, The Swedish Quality Register (SWEQR) Study Group, Allan Abbott, Magnus Ekström, Magnus Forssblad, Peter Fritzell, Åsa Jonsson, Mikael Landén, Michael Möller, Malin Regardt, Björn Rosengren, Marcus Schmitt-Egenolf, Johanna Vinblad, Annette W-Dahl

**Affiliations:** 1 Health Outcomes and Economic Evaluation Research Group, Department of Learning, Informatics, Management and Ethics, Stockholm Centre for Healthcare Ethics, KarolinskaInstitutet, Stockholm, Sweden; 2 Equity and Health Policy Research Group, Department of Global Public Health, Karolinska Institutet, Stockholm, Sweden; 3 Centre for Health Policy, University of Melbourne, Melbourne, Australia; 4 Office of Health Economics, London, UK; 5 City University of London, London, UK; 6 Swedish Arthroplasty Register, Gothenburg, Sweden; 7 Department of Orthopaedics, Institute of Clinical Sciences, Sahlgrenska Academy, University of Gothenburg, Gothenburg, Sweden

**Keywords:** EQ-5D, EQ VAS, Experience-based values, Patient valuation, Swedish National Quality Registers, Health state valuation

## Abstract

**Background:**

The EQ VAS component of the EQ-5D questionnaire has been used to assess patients’ valuation of their own health besides its use for self-reporting of overall health status. The objective of the present study was to identify patients’ valuation of EQ-5D-3L health states using the EQ VAS in different patient groups over time and in comparison to the general population.

**Methods:**

Data were obtained from patients from nine National Quality Registers (*n* = 172,070 patients) at baseline and at 1-year follow-up and compared with data from the general population (*n* = 41,761 participants). The correlation between EQ VAS scores and EQ-5D-3L index based on the Swedish experience-based VAS value set was assessed. Ordinary least squares (OLS) regression models were used to determine the association between EQ-5D-3L dimensions and EQ VAS valuation.

**Results:**

EQ VAS scores showed consistency with severity of health states both at baseline and at 1-year follow-up in the nine selected EQ-5D-3L health states. The regression models showed mostly consistent decrements by severity levels in each dimension at both time points and similar to the general population. The dimension mainly associated with inconsistency was the self-care severity level three. Problems in the anxiety/depression dimension had the largest impact on overall health status in most of the patient groups and the general population.

**Conclusion:**

The study has demonstrated the important role EQ VAS can play in revealing patients’ valuation of their health and showed the variation in valuation of EQ-5D-3L dimensions and levels of severity across different patient groups.

**Supplementary Information:**

The online version contains supplementary material available at 10.1186/s12955-023-02115-z.

## Background

EQ-5D is among the most commonly used generic health-related quality of life questionnaires globally [1]. It has been used to assess health status, as an outcome measure in economic evaluations, in health surveys among the general population and increasingly in routine data collection as part of clinical/health care [2]. The questionnaire has two components: a five-dimension descriptive system and a second component containing the EQ VAS [3]. The five dimensions in the descriptive system are mobility, self-care, usual activities, pain/discomfort and anxiety/depression. The EQ VAS component contains a scaled vertical line ranging from 0 (‘the worst health you can imagine’) to 100 (‘the best health you can imagine’) where respondents rate their overall health status [4, 5]. The questionnaire is available in three-level (EQ-5D-3L) and five-level (EQ-5D-5L) versions for use in adults. The three severity levels in the EQ-5D-3L questionnaire are ‘no problem’, ‘some/moderate problems’ and ‘unable to/extreme problems’. Individuals who report no problems in all the five dimensions of EQ-5D-3L are described to have a health state of 11111, while those with some/moderate problems across the dimensions have a health state of 22222 and 33333 for ones with severe problems across the dimensions [4]. The five severity levels in the EQ-5D-5L are ‘no problems’, ‘slight problems’, ‘moderate problems’, ‘severe problems’ and ‘unable to/extreme problems’ [5].

In summarizing responses to the EQ-5D questionnaire into a single index, various formulas/algorithms termed value sets are employed in different settings [4]. Value sets incorporate the preferences of respondents, reflecting their views about the relative importance of severity levels under each EQ-5D dimension. In the development of value sets for the EQ-5D-3L in different settings, the time trade-off (TTO) and visual analogue scale (VAS) valuation methods have been employed [4]. In valuations through the TTO, respondents are asked to compare living in a specific health state for specific period of time, often 10 years, with living for shorter duration with full health. Through iterations with different durations in full health, the point at which respondents are indifferent describe their values for the specified health state [6, 7]. Valuation using the TTO has also been presented by asking respondents to indicate the duration of time in full health they consider to be equal to living in their current health [8–12].

VAS is used to value health states; whereas the EQ VAS is used to measure overall self-reported health. EQ VAS was introduced as a warm-up task in valuation studies using the VAS, and subsequently became recognised as a useful way of capturing overall self-reported health [13]. Both the EQ VAS and VAS valuations make use of a vertical scale anchored between 0 as the ‘worst imaginable health’ and 100 as the ‘best imaginable health’ indicating that 0 doesn’t equal death [4, 5]. VAS (EQ-5D VAS) is used in the valuation of a number of described health states which may be hypothetical to the respondent [14]. Unlike in EQ VAS for self-reporting of health, in VAS valuations anchoring dead at zero, which allows worse than death states to have values less than zero, is commonly performed [15]. EQ VAS has also been recognised to present a means by which valuations of own (experienced) health of the general population as well as patients can be summarised, which has been shown in several studies [8, 9, 16–19].

Arguments for and against VAS as a valuation method have been made. Some consider that VAS does not have a theoretical basis and that it is not a choice-based method unlike other valuation approaches such as TTO and standard gamble (SG) [20, 21]. VAS not being a choice-based method has been criticized for not allowing respondents the trade-off which is argued as important for valuation methods to be used in economic evaluation. In addition, the fact that VAS valuation doesn’t incorporate uncertainty which is considered a desired attribute in valuation is criticized and has been associated with lower/downward valuations compared to TTO and SG [20, 22, 23]. Another criticism raised concerns the middle point bias/end-aversion bias where respondents avoid the lower and upper ends of the VAS scale [20, 21]. However, arguments for the use of VAS in health state valuation challenge the above views and indicate that empirical performance of valuation methods should be focused on in assessing their performance in health state valuation [24]. The practical role of VAS in health state valuation was also demonstrated in a recent scoping review which showed its use in different research areas including clinical studies [25].

The perspective respondents take in valuation studies could be experience-based or hypothetical. In a hypothetical perspective, respondents value a sub-set of health states described in the EQ-5D instrument, which they may never have experienced themselves and are asked to imagine experiencing. Most EQ-5D-3L and EQ-5D-5L value sets were developed through members of the general public being asked to take this hypothetical perspective [4, 5]. The arguments toward the use of hypothetical perspective point to the fact that resource allocation in society should be made by the general population [22, 26]. In contrast, experience-based valuations entail respondents valuing their own health [27]. It has been employed in the development of a number of EQ-5D-3L [8, 17, 19] and EQ-5D-5L [9, 18] value sets as well as health state valuations among patients [16, 28, 29]. Arguments for the use of experience-based valuations concern the idea that individuals experiencing specific condition/health state are the best sources of information regarding that [22, 26].

EQ VAS has been employed to assess both general population values and patient valuations of their health in different studies. In using EQ VAS data to create experience-based values, EQ VAS scores are modelled based on the levels of severity reported in the five EQ-5D dimensions [16, 28, 30–34]. Studies using this approach include a study in the UK comparing VAS valuations in the general population and patients with different conditions [30]; a comparison of valuations of patients undergoing total hip arthroplasty with the general population [16]; and exploration of value sets among patients undergoing total knee replacement, both in the UK [31]. Another study used EQ VAS to establish values of patients with non-specific low-back pain compared to the general population in the Netherlands [32]; and valuations among patients with different medical conditions in the UK [33, 34]. A study in Sweden comparing patient value sets from individuals who underwent total hip arthroplasty with general population ones [28] also employed the EQ VAS. However, in the valuation of health states using the EQ VAS a literature gap remains in comparing how valuation varies across different EQ-5D-3L dimensions within patient groups over time and across different patient groups.

In Sweden, there are approximately 100 National Quality Registers (NQRs) which collect clinical data on individual patients with the aim of improving the quality of health care provided to them. As part of this, the data in the registers are employed in research. In about 40 of the registers, data on the EQ-5D questionnaire is collected routinely, including patients’ EQ VAS [35]. With their large sample sizes in different patient groups, these registers provide useful data sources to assess patients’ valuations of own health states.

Studying the NQR data in investigating the characteristics of patient valuations using the EQ VAS in different patient groups and over time could contribute to the literature on the importance of different EQ-5D-3L dimensions to patients in influencing their EQ VAS score. Such a study will also provide information on the relative importance of different dimensions in various patient groups and how this compares to that of the general population. Overall, the study could provide comprehensive information on the role of the EQ VAS in patient valuation of health states. Accordingly, the objective of the present study was to identify patients’ valuation of EQ-5D-3L health states using the EQ VAS in different patient groups over time and in comparison to the general population.

## Methods

### Study design

EQ VAS data of patients from nine Swedish NQRs at baseline and 1-year follow-up were assessed and compared with the general population. The present study forms part of the research project described in a study protocol published elsewhere, containing detailed information on the background and the NQRs in the study [36]. It follows up on a previous study in the project [37].

### Data

Data from nine Swedish NQRs and the general population were employed in the study. The nine registers were selected from those collecting EQ-5D data to include different types of diseases and conditions to make comparisons possible. The availability of EQ VAS data covering patients in the registers; availability of follow-up data as well as willingness of registers to be part of the research project also determined the selection of registers included in the study. The NQRs include six intervention-based registers covering spine surgery, hip, knee, ankle replacement, cruciate ligament injury treatment, and first-line osteoarthritis (Better management of patients with OsteArthritis (BOA)) treatment, and three diagnosis-based registers covering heart failure, respiratory failure, and bipolar disorders. Data on patient-reported outcomes on the EQ-5D-3L questionnaire were retrieved from the NQRs as well as the general population in the study.

EQ VAS data both at baseline and at 1-year follow-up were included to capture patient valuations in different circumstances which could provide clearer information on how one’s valuation of health changes in relation to the change in the disease/condition over time.

The general population data used in the comparison were based on the population survey data of individuals in Scania Region in 2004 and Region Stockholm in 2006, which are generally representative of the Swedish population. Living conditions and self-reported health, which included the EQ-5D-3L questionnaire, were assessed [8, 38–40]. The Swedish experience-based EQ-5D-3L value sets were developed using this survey data [38, 40]. In the present study, data of 41,761 respondents with complete data on the five EQ-5D-3L dimensions and EQ VAS were included [8].

In the present study, in calculating EQ-5D-3L index the Swedish experience-based EQ-5D-3L VAS value set was used [8]. This value set was developed in the above described survey where members of the general population valued their own health states through both TTO and VAS methods [8].

### Sample size

The study included records of patients with complete data on demographic and EQ-5D-3L data. Data of patients with complete data on EQ VAS score were included at baseline and 1-year follow-up. A total of 172,070 patient records are available from the nine NQRs with data on EQ VAS score and data from a total of 41,761 participants from the general population. A detailed description of the sampling procedure is presented in Table S1.

### Data analysis

Descriptive analyses on the frequency and proportions of demographic characteristics and problems reported in the five EQ-5D-3L dimensions in each patient group and the general population were performed. The mean and standard deviations of patients’ EQ VAS scores for nine selected EQ-5D-3L health states were calculated in each patient group at baseline and at 1-year follow-up and for the general population. Six of the nine EQ-5D-3L health states were selected as they were common across different patient groups facilitating comparisons of valuations. In addition, the health states 11111, 22222 and 33333 were also selected to compare valuations of full, moderate, and worst health states across patient groups. Considering the association of problems reported on the EQ-5D-3L dimensions with the EQ VAS, the EQ VAS score has been used in the present study as a valuation of the health states reported on the EQ-D-3L dimensions. Owing to the broader construct of EQ VAS than the EQ-5D descriptive system [13], a person reporting a health state 11111 could still report an EQ VAS score of < 100.

The correlation between EQ VAS score and the EQ-5D-3L index based on the Swedish experience-based EQ-5D-3L VAS value set [8], in each register and the general population, was analysed using Spearman’s rank correlation. The correlation between changes in EQ VAS score and EQ-5D-3L index was also performed additionally. Spearman’s rank correlation is used as it does not require normality of distribution of the variables [41]. The resulting correlation coefficients were interpreted using the cut-off values of 0.00 to 0.19 as very weak, 0.20 to 0.39 as weak, 0.40 to 0.69 as moderate, 0.70 to 0.89 as strong, and 0.90 to 1.00 as very strong [42].

Ordinary least squares (OLS) models were used to assess the predictive effect of EQ-5D-3L dimensions on EQ VAS score at baseline and at 1-year follow-up in the nine NQRs. The regression models were performed both in the unadjusted form and adjusted for sex and age groups. The results of the regression analyses were compared with that of the general population in terms of the estimates in each of the EQ-5D-3L dimensions and the severity levels of the problems reported in each dimension. In assessing the face validity of the models, inconsistency was defined as the occurrence of a lower magnitude of decrement for a specific severity level in an EQ-5D-3L dimension than the decrement of a milder level of severity (e.g. if self-care level 3 has a lower decrement than self-care level 2, it is considered an inconsistency). Further OLS models were also performed using the pooled patient data at baseline and 1-year follow-up to assess how patient groups are associated with VAS valuation. In addition to OLS models, multilevel models (two-level random slope and random intercept models) were also performed. A p-value of 0.05 was used as a cut-off for statistical significance. In order to assess the overall translatability of the findings in the analysis here, regression models of baseline and 1-year follow-up EQ-5D-5L data were conducted. The analyses were performed using R version 3.5.0/3.5.1 and SAS version 9.4.

## Results

### Demographic characteristics

The mean age of the patients in the nine registers ranged from about 30 in cruciate ligament injury to older than 73 years among respiratory failure patients, while the mean age was 45.5 years in the general population. In most of the registers, the majority of patients were in the age groups of 50s to 70s while from 30s to 50s in the general population. Women constituted the majority in five of the registers, similar to the general population, except in ankle, cruciate ligament injury and heart failure registers (Table 1).

**Table 1:** Demographic characteristics of patients in the 9 National Quality Registers (NQRs) and in the general population

Variable	Intervention-based registers						Diagnosis-based registers			General population
	**Spine**	**Hip**	**Knee**	**Ankle**	**Cruciate ligament**	**BOA**	**Heart failure**	**Respiratory failure**	**Bipolar**	
	*n* = 44,196	*n* = 90,658	*n* = 16,324	*n* = 668	*n* = 8,155	*n* = 6,690	*n* = 1,044	*n* = 725	*n* = 3,610	*n* = 41,761
	**%**	**%**	**%**	**%**	**%**	**%**	**%**	**%**	**%**	**%**
**Age in years** [mean (SD)]	58.4 (4.3)	68.5 (10.3)	68.9 (8.7)	63.0 (11.7)	29.7 (10.0)	65.8 (9.0)	71.8 (11.1)	73.2 (7.7)	58.4 (15.3)	45.5 (14.9)
**Age group**										
30	3.6	0.1	0.01	1.5	59.2	0.1	0.2	-	15.0	17.1
30–39	9.9	0.7	0.1	3.0	21.2	0.5	0.5	0.1	18.8	20.4
40–49	16.2	3.7	1.7	7.5	15.3	4.3	3.1	0.1	20.7	20.8
50–59	18.5	13.0	13.1	21.1	4.1	17.3	10.6	4.6	18.9	21.6
60–69	24.1	33.4	35.6	33.5	0.3	43.8	22.6	26.2	17.5	14.9
70–79	21.7	35.2	38.6	30.2	0.01	28.3	35.8	47.7	7.8	4.8
80 +	6.1	13.9	11.0	3.1	-	5.6	27.2	21.2	1.3	0.4
**Sex**										
Women	51.1	56.5	57.5	44.8	44.7	70.8	31.9	60.6	64.1	56.2

*BOA* Better management of patients with OsteoArthritis, *SD* standard deviation

### Problems reported on the EQ-5D-3L dimensions and EQ VAS score

At baseline, the highest proportion of any problems (level 2 or level 3) were reported in the pain/discomfort dimension among patients in most of the registers and in the general population. Among patients in the respiratory failure and bipolar disorder registers the dimensions with the highest proportions of problems reported were mobility and anxiety/depression respectively. The highest proportions of severe (level 3) problems were also reported in the pain/discomfort dimension across most registers and in the general population. In patients with respiratory failure and bipolar disorder the highest proportions of severe problems were reported in the usual activities and anxiety/depression dimensions respectively (Table 2).

**Table 2:** Problems reported on the EQ-5D-3L dimensions by patients in the 9 National Quality Registers (NQRs) and in the general population

**Patient group in NQRs/ general population**		**Severity level**	**EQ-5D-3L dimension**										**EQ VAS score** Mean (SD)	
			**Baseline**					**1-year follow-up**						
			**Mobility**	**Self-care**	**Usual activities**	**Pain/** **discomfort**	**Anxiety/** **depression**	**Mobility**	**Self-care**	**Usual activities**	**Pain/** **discomfort**	**Anxiety/** **depression**	**Baseline**	**1-year follow-up**
			**%**	**%**	**%**	**%**	**%**	**%**	**%**	**%**	**%**	**%**		
**Intervention-based registers**	**Spine** (*n* = 44,196)	Level 1	15.3	**79.7**	27.7	0.6	41.9	**57.7**	**91.7**	**65.9**	23.0	**62.4**	47.8 (22.1)	67.9 (22.5)
		Level 2	**82.7**	19.1	**53.4**	49.5	**52.1**	42.1	7.8	29.8	**63.2**	33.4		
		Level 3	2.0	1.2	19.0	**49.9**	6.0	0.3	0.5	4.4	13.8	4.2		
	**Hip** (*n* = 90,658)	Level 1	8.1	**77.7**	40.1	1.5	**59.7**	**61.7**	**92.4**	**78.0**	45.5	**78.4**	56.3 (22.2)	76.7 (20.0)
		Level 2	**91.6**	21.3	**50.1**	**57.9**	37.1	38.1	7.0	20.0	**50.1**	20.1		
		Level 3	0.3	1.0	9.8	40.5	3.3	0.1	0.6	2.0	4.4	1.5		
	**Knee** (*n* = 16,324)	Level 1	11.6	**93.4**	**53.4**	1.8	**65.4**	**62.8**	**95.1**	**77.8**	36.1	**78.6**	64.6 (22.1)	76.2 (19.4)
		Level 2	**88.1**	5.8	41.3	**63.3**	32.2	37.1	4.4	20.6	**58.7**	19.7		
		Level 3	0.3	0.9	5.3	35.0	2.4	0.2	0.6	1.7	5.2	1.7		
	**Ankle** (*n* = 668)	Level 1	3.9	**88.3**	37.7	1.1	**57.5**	36.7	**91.5**	**63.9**	24.3	**71.4**	55.8 (21.4)	70.2 (18.9)
		Level 2	**94.5**	10.8	**49.3**	**49.7**	38.2	**62.9**	7.5	32.2	**65.6**	26.2		
		Level 3	1.7	0.9	13.0	49.3	4.3	0.5	1.1	3.9	10.2	2.4		
	**Cruciate ligament** (*n* = 8,155)	Level 1	**67.0**	**97.4**	**53.6**	15.2	**49.6**	**86.9**	**98.9**	**79.0**	35.0	**65.2**	62.8 (23.0)	74.5 (19.9)
		Level 2	32.7	2.1	37.4	**79.0**	44.9	13.1	0.8	19.4	**61.9**	31.5		
		Level 3	0.3	0.5	9.0	5.7	5.5	0.1	0.3	1.6	3.1	3.3		
	**BOA** (*n* = 6,690)	Level 1	42.9	**96.0**	**75.6**	2.4	**67.0**	**53.3**	**96.2**	**80.1**	9.8	**71.1**	68.3 (18.6)	70.4 (18.7)
		Level 2	**57.0**	3.7	23.0	**87.3**	31.7	46.6	3.4	19.2	**82.6**	27.9		
		Level 3	0.1	0.4	1.4	10.3	1.2	0.1	0.4	0.8	7.6	1.1		
**Diagnosis-based registers**	**Heart failure** (*n* = 1,044)	Level 1	**60.6**	**91.9**	**72.6**	**47.7**	**60.3**	**59.3**	**91.3**	**72.8**	**49.2**	**62.7**	65.4 (18.9)	67.4 (18.8)
		Level 2	39.2	7.7	24.0	47.5	36.6	40.2	8.0	24.2	43.5	34.4		
		Level 3	0.2	0.5	3.5	4.8	3.2	0.5	0.8	3.0	7.3	2.9		
	**Respiratory failure** (*n* = 725)	Level 1	22.8	**62.3**	25.9	29.0	42.1	17.0	**53.4**	19.5	23.9	37.5	51.1 (21.3)	49.1 (20.4)
		Level 2	**75.6**	32.4	**53.8**	**57.7**	**49.0**	**79.0**	38.5	**50.5**	**60.0**	**52.3**		
		Level 3	1.7	5.2	20.3	13.4	9.0	4.0	8.1	30.1	16.1	10.2		
	**Bipolar** (*n* = 3,610)	Level 1	**85.7**	**94.9**	**70.3**	**53.0**	38.4	**82.5**	**93.7**	**69.5**	**50.6**	39.6	66.3 (21.1)	67.2 (20.1)
		Level 2	14.1	4.7	26.0	40.3	**50.6**	17.0	5.8	26.5	41.6	**49.8**		
		Level 3	0.2	0.4	3.7	6.8	11.0	0.5	0.4	4.1	7.8	10.5		
**General population** (*n* = 41,761)		Level 1	**91.3**	**98.6**	**91.6**	**53.5**	**68.4**	-	-	-	-	-	79.5 (18.3)	-
		Level 2	8.6	1.1	7.3	42.7	29.0	-	-	-	-	-		
		Level 3	0.1	0.4	1.1	3.8	2.7	-	-	-	-	-		

*BOA* Better management of patients with OsteoArthritis, *SD* standard deviation; level 1: no problem; level 2: some/moderate problems; level 3: confined to bed/unable to/ extreme problems; the level with the highest proportion in each group is shown in bold

At 1-year follow-up the proportion of problems reported across the EQ-5D-3L dimensions decreased in almost all the patient groups. Pain/discomfort remained the dimension where the highest proportions of any problems were reported in most registers. In respiratory failure and bipolar disorder registers, the highest proportions of problems were reported in the mobility and anxiety/depression dimensions respectively. The most frequent severe problems were also reported in the pain/discomfort dimension in most registers while anxiety/depression dimension constituted the most frequent problems in cruciate ligament injury and bipolar disorder registers (Table 2).

**Fig. 1:**
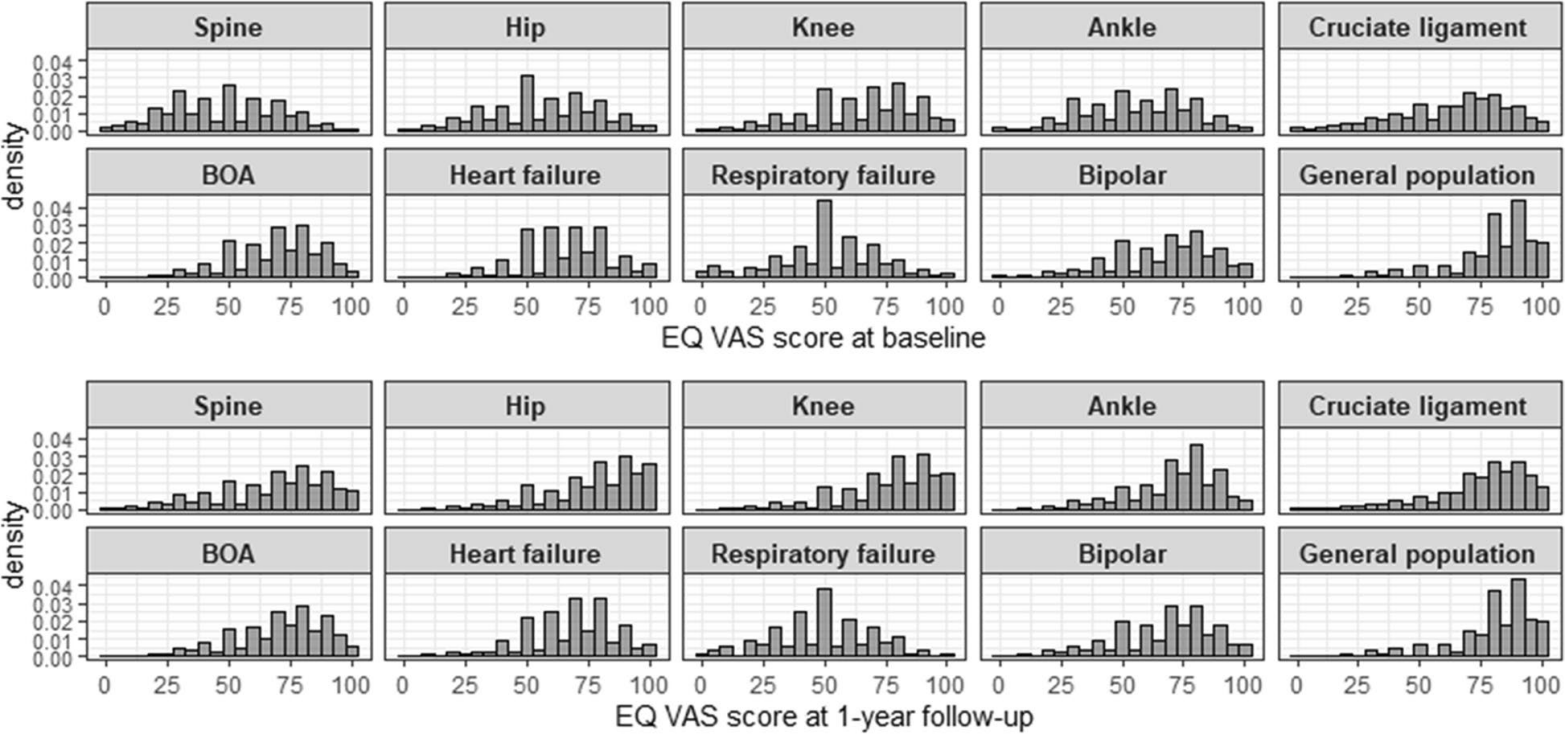
Distribution (density plot) of EQ VAS score by patient group at baseline and at 1-year follow-up and in the general population (general population data, based on a cross-sectional survey) [BOA: Better management of patients with OsteoArthritis]

At baseline, mean EQ VAS score ranged from 47.8 among patients in the spine register to 68.3 among patients in the BOA register, while it was 79.5 in the general population. At 1-year follow-up mean EQ VAS score ranged from 49.1 among respiratory failure patients to 76.7 in patients who underwent hip replacement. Mean EQ VAS scores showed increases between baseline and 1 year in almost all the patient groups with the exception of patients with respiratory failure. The increases were the highest in most of the intervention-based registers (Table 2). Figure 1 shows the distribution of EQ VAS scores across the patient groups at baseline and at 1-year follow-up and that of the general population.

### Mean EQ VAS scores for selected health states

In total, 204 and 202 distinct health states were reported among the nine patient groups at baseline and at 1-year follow-up respectively. In the general population, 152 distinct health states were reported (Table 3).

**Table 3:** Mean EQ VAS values among nine selected EQ-5D-3L health states across patient groups and the general population, baseline and 1-year follow-up

**Time**	**Health** **state**	**Mean EQ VAS scores (standard deviation)**									
		**Intervention-based registers**						**Diagnosis-based registers**			
		**Spine**	**Hip**	**Knee**	**Ankle**	**Cruciate ligament**	**BOA**	**Heart failure**	**Respiratory failure**	**Bipolar**	**General pop**
**Baseline**	11111	78.3 (17.3)	75.9 (21.4)	81.7 (15.7)	82.5 (3.5)	78.3 (18.2)	87.2 (12.1)	76.4 (15.7)	68.8 (23.2)	82.4 (14.0)	88.8 (9.7)
	11121	69.8 (16.9)	73.5 (17.3)	80.8 (15.2)	73.9 (20.7)	72.8 (19.4)	78.1 (14.3)	72.1 (16.4)	61.9 (24.1)	77.9 (14.0)	82.5 (11.6)
	21121	64.0 (17.4)	67.6 (17.8)	73.1 (17.9)	69.3 (16.1)	64.0 (20.7)	71.2 (16.1)	66.8 (13.2)	60.6 (15.6)	75.8 (12.2)	72.0 (17.0)
	21122	56.3 (17.0)	59.8 (16.9)	63.8 (17.8)	61.2 (17.5)	54.5 (21.9)	62.9 (16.2)	57.1 (16.7)	57.3 (23.0)	58.8 (18.9)	59.7 (18.2)
	21221	57.2 (17.7)	61.9 (18.0)	67.2 (18.4)	60.6 (17.9)	60.6 (22.5)	63.8 (16.0)	57.7 (14.7)	52.6 (19.0)	70.8 (14.9)	59.8 (18.7)
	21222	49.8 (16.6)	54.2 (16.9)	57.9 (18.3)	57.4 (13.5)	51.9 (20.7)	54.6 (16.2)	52.0 (15.2)	48.7 (14.7)	56.0 (14.5)	51.7 (17.7)
	21232	39.8 (19.6)	45.1 (20.6)	49.4 (21.5)	47.6 (19.4)	43.4 (22.5)	49.7 (19.3)	42.7 (14.9)	51.4 (26.1)	46.8 (17.7)	38.4 (16.4)
	22222	45.8 (17.6)	51.7 (17.2)	50.1 (18.1)	52.0 (5.7)	46.6 (20.1)	51.2 (17.2)	51.5 (24.0)	50.3 (17.8)	53.0 (18.6)	44.4 (17.3)
	33333	13.3 (20.4)	22.3 (26.2)	-	-	-	-	-	-	-	36.7 (45.9)
	Total number of health states	168	169	108	51	103	78	69	92	102	152
**1-year follow-up**	**Heaath state**	**Mean EQ VAS scores (standard deviation)**									
		**Intervention-based registers**						**Diagnosis-based registers**			
		**Spine**	**Hip**	**Knee**	**Ankle**	**Cruciate ligament**	**BOA**	**Heart failure**	**Respiratory failure**	**Bipolar**	**-**
	11111	89.2 (10.6)	89.9 (11.4)	88.8 (11.9)	83.6 (11.1)	85.5 (14.4)	89.2 (11.4)	78.5 (14.3)	64.2 (23.1)	82.5 (12.0)	-
	11121	79.5 (12.3)	81.5 (12.3)	81.9 (12.4)	82.7 (8.5)	78.9 (15.1)	80.0 (12.2)	75.3 (11.5)	74.1 (14.1)	79.2 (12.7)	-
	21121	69.2 (14.8)	73.4 (14.8)	73.6 (14.7)	73.2 (15.9)	71.4 (17.3)	69.7 (15.0)	69.8 (14.8)	64.5 (15.6)	76.2 (15.4)	-
	21122	60.2 (15.1)	63.5 (15.4)	63.5 (16.1)	68.0 (11.2)	59.8 (20.6)	60.0 (14.2)	59.5 (16.1)	50.9 (24.7)	66.7 (15.3)	-
	21221	59.5 (15.9)	64.0 (16.1)	66.5 (16.5)	65.7 (17.3)	64.6 (17.7)	61.1 (16.0)	59.8 (16.6)	52.5 (17.5)	62.9 (11.9)	-
	21222	52.5 (15.1)	55.2 (15.7)	57.2 (15.9)	59.4 (14.1)	55.8 (18.7)	53.3 (14.7)	54.4 (14.6)	49.4 (14.9)	54.5 (15.1)	-
	21232	39.1 (17.5)	40.4 (17.7)	45.4 (20.5)	47.4 (20.3)	42.7 (19.0)	44.3 (19.0)	55.6 (14.7)	45.8 (6.2)	43.5 (15.1)	-
	22222	48.2 (15.8)	51.6 (16.6)	52.4 (17.4)	56.9 (10.7)	41.7 (18.9)	50.4 (13.3)	42.0 (20.5)	43.7 (16.6)	53.5 (13.9)	-
	33333	16.8 (20.5)	26.3 (31.6)	39.8 (42.0)	-	-	-	-	-	-	-
	Total number of health states	159	184	125	56	82	76	72	90	111	-

*BOA* Better management of patients with OsteoArthritis

Both at baseline and at 1-year follow-up, mean EQ VAS scores of the nine selected EQ-5D-3L health states were higher for milder health states and lower among more severe health states. The increase in EQ VAS scores of the health state 11111 and 11121 from baseline to respective health states at 1-year follow-up was higher in most of the intervention-based registers than in diagnosis-based registers. For both health states, at baseline, the EQ VAS scores in most patient groups were lower than in the general population. At 1-year follow-up, the EQ VAS scores increased to be closer to general population. The differences in EQ VAS score from baseline to 1-year follow-up, in the remaining seven health states, were generally lower than that of the health states 11111 and 11121 (Table 3).

The health state 22222 had mean EQ VAS scores at baseline that varied from 45.8 among spine patients to 53.0 among bipolar patients. At 1-year follow-up it varied from 41.7 in cruciate ligament injury patients to 56.9 in patients in ankle register. Meanwhile, 22222 had a mean EQ VAS score of 44.4 in the general population (Table 3).

### Correlation between EQ VAS score and EQ-5D-3L index

EQ VAS scores reported by patients in each register and the general population were correlated with EQ-5D-3L indices based on the Swedish experience-based EQ-5D-3L VAS value set. At baseline almost all patient groups and the general population showed moderate levels of correlation with the EQ-5D-3L index. At 1-year follow-up, correlation coefficients in all patient groups showed increase from baseline. Correlation coefficients among patients in the spine and hip registers were strong. In all the remaining registers moderate levels of correlation were found (Table 4). The results of the correlation analysis between changes in EQ VAS score and changes in EQ-5D-3L index showed low to moderate levels of correlation across the different patient groups (Table S2).

**Table 4:** Spearman’s rank correlation coefficient between EQ VAS score and EQ-5D-3L index based on Swedish experience-based EQ-5D-3L VAS value set

**Register/ general population**		**Spearman’s correlation**			
		**Baseline**		**1-year follow-up**	
		**Coefficient**	***P*-value**	**Coefficient**	***P*-value**
**Intervention-based**	**Spine**	0.522	< 0.0001	0.769	< 0.0001
	**Hip**	0.441	< 0.0001	0.702	< 0.0001
	**Knee**	0.419	< 0.0001	0.644	< 0.0001
	**Ankle**	0.475	< 0.0001	0.638	< 0.0001
	**Cruciate ligament**	0.428	< 0.0001	0.564	< 0.0001
	**BOA**	0.499	< 0.0001	0.640	< 0.0001
**Diagnosis-based**	**Heart failure**	0.520	< 0.0001	0.562	< 0.0001
	**Respiratory failure**	0.400	< 0.0001	0.539	< 0.0001
	**Bipolar**	0.619	< 0.0001	0.650	< 0.0001
**General population**		0.596	< 0.0001	-	-

*BOA* Better management of patients with OsteoArthritis

### Regression models of the baseline data

The OLS models of the baseline data showed that EQ-5D-3L dimensions predicted EQ VAS score in a mostly consistent way in most of the NQRs and in the general population data. In seven of the nine NQRs and in the general population, the main inconsistency was in the self-care severity level three with lower decrement than in level two. Additionally, inconsistency in the mobility dimension was shown in the knee and heart failure registers and in the general population. In the respiratory failure and bipolar registers, the inconsistency was in the mobility dimension (Table 5, Fig. S1).

**Table 5:**
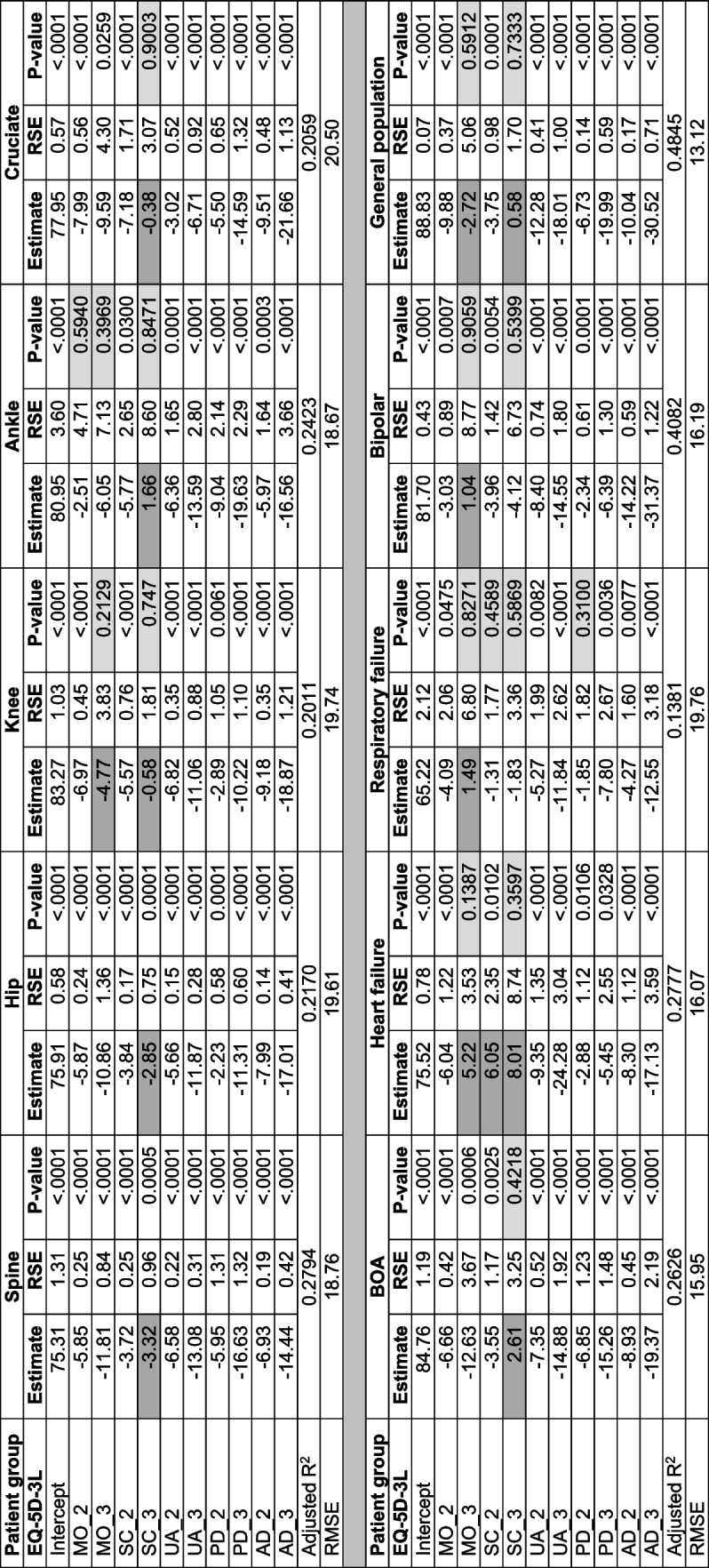
Ordinary least squares regressions in the 9 registers at baseline and in the general population

*BOA* Better management of patients with OsteoArthritis, *RMSE* Root mean square error, *RSE* Robust standard error; Darker shades under estimate columns show inconsistency in decrement; Lighter shades in the *P*-value columns show non-statistically significant estimates;

In all except three registers—spine, ankle and heart failure—and in the general population—the highest decrements in EQ VAS score were in the anxiety/depression dimension level three. In the spine and ankle registers level three in the pain/discomfort dimension and the usual activities dimension in the heart failure register had the largest decrements in EQ VAS score (Table 5). The adjusted R squared values ranged from about 14% in respiratory failure patients to about 41% in bipolar patients, and was 48% in the general population. The OLS models at baseline adjusted for sex and age group showed similar results to the unadjusted models in most of the registers and in the general population (Table S3).

In the two-level model of the data at baseline, findings similar to the OLS models were shown. Accordingly, self-care level three is the dimension with inconsistency in all of the registers and in the general population. Across dimensions the highest decrement was in the anxiety/depression in all the registers and in the general population except for the spine register (pain/discomfort dimension) (Table S4). The adjusted version of the models showed results similar to the unadjusted models (Table S5). Comparing the OLS and multilevel models, both unadjusted and adjusted models showed that the multilevel models had estimates with narrower 95% confidence interval than the OLS models (Fig. S1-S4).

### Regression (OLS) models of the one-year follow-up data

In the OLS models based on the 1-year follow-up data across the registers, the decrements in all the EQ-5D-3L dimensions showed consistency with severity levels in four of the registers. The registers with any occurrence of inconsistency in the decrements included the spine, ankle, cruciate, heart failure and bipolar registers. The inconsistency in these registers were mainly related to self-care dimension in spine, ankle and cruciate registers and mobility dimensions in the heart failure and bipolar registers. Additional inconsistency in the mobility dimension was also shown in the cruciate ligament injury register.

In most of the registers and in the general population, the highest decrements in EQ VAS score were observed in the anxiety/depression dimension. The exceptions were spine and ankle registers with pain/discomfort and mobility dimensions having the highest decrements respectively (Table 6, Fig. S6). The adjusted R squared values of the models at 1-year follow-up ranged from about 30% in patients with respiratory failure to 60% in the spine register (Table 6). The OLS models at 1-year follow-up adjusted for sex and age group showed similar results to the unadjusted models (Table S6).

**Table 6:**
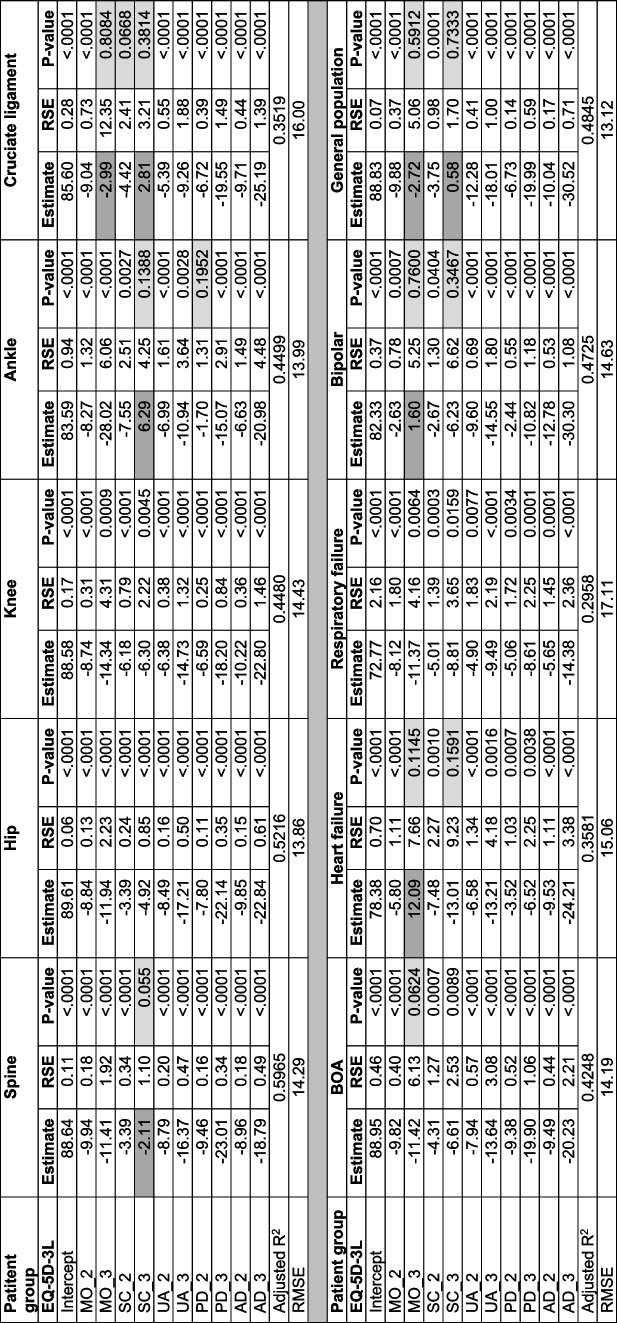
Ordinary least squares regressions in the 9 registers at 1-year follow-up and in the general population

*BOA* Better management of patients with OsteoArthritis, *RMSE* Root mean square error, *RSE*: Robust standard error; Darker shades under estimate columns show inconsistency in decrement; Lighter shades in the *P*-value columns show non-statistically significant estimates

In the two-level model, two of the registers (hip and respiratory failure) had estimates with consistency in all dimensions. In all the other registers inconsistencies were related to the self-care dimension. In terms of the decrements of EQ VAS score, anxiety/depression dimension had the largest decrement in eight of the nine registers and in the general population data. In the spine register, the largest decrement was shown in the pain/discomfort dimension (Table S7). The models adjusted for sex and age groups, had similar findings to the unadjusted models (Table S8). The 95% CIs of the estimates, both in the unadjusted and adjusted models, were narrower compared to the estimates of the OLS models (Fig. S5-S8).

### Findings of additional analyses

Results of the analysis of the pooled data with patient groups as independent variables showed that the overall association between EQ-5D-3L dimensions and The EQ VAS remained stable. Almost all the patient groups showed association with EQ VAS score with different levels of decrement or increment in comparison to the general population (Tables S9, S10).

The OLS models based on EQ-5D-5L data of the two patient groups (BOA and hip) demonstrated an overall comparable findings to those of the findings in the main analysis based on the EQ-5D-3L data (Table S11).

## Discussion

In the present study, patient valuations of their health states using the EQ VAS was explored. Valuations of nine selected EQ-5D-3L health states showed general consistency by severity. Similarly, moderate to strong correlations were found between EQ VAS valuations based on modelled EQ VAS data and the EQ-5D-3L index. A change in the EQ VAS scores of the same health states over time and differences across patient groups was also observed. Models of EQ VAS score regressed on EQ-5D-3L dimensions showed mostly consistent decrements by severity level in each dimension both at baseline and at 1-year follow-up.

Patients’ values, across nine selected EQ-5D-3L health states, had good face validity: poor health states had lower values than mild health problems, suggesting that these methods provide a reasonable means of broadly reflecting how good or bad patients consider health states to be. The consistency of EQ VAS scores of health states with severity was also reported by a study of the general population on data from 15 countries [43]. A number of common health states like in the present study were also reported preoperatively and postoperatively in a study of a knee arthroplasty population in the UK [31].

In the EQ VAS scores of the same health states at baseline and at 1-year follow-up, relatively larger increases were observed in valuations of many of the health states from the intervention-based registers than in the diagnosis-based registers. This could be associated with the mainly surgical interventions employed in most of the intervention-based registers relieving pain and mobility problems which are common among such patients [44]. In relation to this, the results also seem to support the face validity of using EQ VAS as patient valuations. The differences in EQ VAS scores of the same health states across patient groups and over time could be associated with the broader nature of EQ VAS compared to the EQ-5D-3L, hence capturing broader aspects of health than the EQ-5D dimensions. This was also reported by a study in the UK patient-reported outcome measures program [13].

The overall consistency between the EQ VAS and the EQ-5D-3L dimensions was also shown in the significant correlations between EQ VAS score and EQ-5D-3L index which increased from baseline to 1-year follow up in all the patient groups. Comparable findings were reported in similar studies among women with cervical pre-cancer (0.51) in the UK [34], and among patients with Parkinson’s disease (0.68) in Spain [45].

The OLS models of EQ VAS regressed on the EQ-5D-3L dimensions both at baseline and 1-year follow-up showed mostly consistent decrements by severity level in each dimension in the different patient groups and the general population. An overall similar finding was reported in a study which compared health state valuation between non-specific low back pain patients and the general population in the Netherlands [32]. Similarly, overall consistent models were reported in previous studies based on preoperative and postoperative data of patients undertaking hip replacement in Sweden [28] and knee replacement in the UK [31]. In addition, studies in the general population conducted through experience-based [8, 9, 18, 19, 46, 47] and hypothetical [48–50] perspectives also showed consistent valuations of EQ-5D health states (3L as well as 5L) through VAS.

In the OLS models, inconsistencies were noted mainly in the self-care dimensions in all the patient groups at baseline and in several patient groups at 1-year follow-up. Inconsistencies in the mobility dimension were also shown among several patient groups at baseline and at 1-year follow-up. Similarly, a study in Sweden exploring valuations among patients undergoing hip replacement the self-care dimension showed inconsistency both in preoperative and postoperative valuations [28]. In another study in the UK among patients in four clinical groups (stroke, low back pain, colposcopic investigation and cytological surveillance), the self-care dimension was found to not be statistically significant in either of the severity levels 2 and 3 [33]. Inconsistencies in the self-care and mobility dimensions were also noted among low back pain patients in the study from the Netherlands [32]. In the present study, the inconsistencies were also noted among in the general population.

One of the possible reasons for the inconsistencies noted in the present study in the self-care and to a certain extent in the mobility dimensions could be the relative importance of the different dimensions depending on whether one is valuing their own health state or a hypothetical health state. This has been discussed in a previous study, based on EQ-5D-3L data from the US, which compared experienced and hypothetical health states where the self-care dimension followed by pain/discomfort were the most important dimensions in the valuation of hypothetical health states. In contrast, usual activities and anxiety/depression dimensions were the most important in experience-based valuations [51]. In relation to this, the study also showed that in the experience-based valuations severity levels 2 and 3 of the mobility and self-care dimensions were closest to each other compared to other dimensions and to hypothetical valuations [51]. This was in line with the findings across the patient groups as well as in the general population in the present study. The findings here show that that the aspects of health important to patients are different from those of the general public who are asked to imagine health problems. This, in turn, could yield different results when measuring effectiveness of interventions depending on whether patients’ own perspectives or imagined health states by the general public are used.

The second related possible explanation for the inconsistencies could be the relatively small number of individuals reporting severity level 3 problems in the self-care and mobility dimensions. In the mobility dimension, the fact that level 3 is presented as ‘confined to bed’ could have contributed to fewer individuals reporting that level. In relation to that, individuals with more severe problems choosing mobility severity level 2 could possibly contribute to lower EQ VAS scores. In a number of studies where inconsistencies in one or both of the dimensions were shown, the relative number of responses falling in severity level 3 were small accounting for 1% or less of the all the problem levels in studies from Sweden and the UK [28, 32–34]. Notably, in the study from the UK among women with low-grade cytological abnormalities (pre-cancer), severity levels 2 and 3 in mobility, self-care, and usual activities dimensions were combined due to very few number of individuals reporting problems in these dimensions [34]. Comparatively, EQ-5D-5L has been shown to provide better discriminatory power between severity levels than EQ-5D-3L [52] and lower ceiling effects the potential implications of which, on valuation, have been discussed [52, 53]. In the context of the present study the categorization of responses in the ‘no problem’ or ‘moderate’ levels, which would otherwise be in between in EQ-5D-5L, could lead to under/over estimation of valuations.

In the present study, anxiety/depression showed the highest decrements in most patient groups at baseline and at 1-year follow-up indicating it to be the most important dimension to patients. A similar finding was also shown in the general population data. In a study based on data from different groups—people with varicose veins, chest pain, chronic obstructive pulmonary disease, irritable bowel syndrome, osteoarthritis, low back pain, elderly women and patients in intensive care unit – anxiety/depression was the dimension with the highest decrement, similar to the present study [30]. Similar findings were also shown in several other studies in Sweden [28, 47], the Netherlands [32] and the UK [16, 31, 33, 34], employing patient valuations of their own health.

The dimensions with the highest decrements remained the same from baseline to 1-year follow up in most of the patient groups. However, in patients from the ankle and heart failure registers, a change in the dimension with the highest decrement with time was shown. In the ankle register, pain/discomfort had the highest decrement at baseline and anxiety/depression at 1-year follow-up. Heart failure patients on the other hand, had the highest decrement in the usual activities dimension at baseline and in the anxiety/depression dimension at 1-year follow-up. This could relate to the change in the relative importance of the different dimensions depending on the disease/condition patients have and how they experienced them before and after intervention/treatment. It is also notable that the dimension with the highest decrement at 1-year follow-up in the two patient groups had become similar to that of the general population in the study.

In the anxiety/depression dimension, the highest decrement was recorded among patients with bipolar disorder both at baseline and at 1-year follow-up. This seems to show the ability of the EQ-5D instrument to indicate the importance of specific dimensions to patients in line with their diagnosis/conditions. Comparably large decrements in the anxiety/depression dimension was noted in a study from the UK [33]. The decrements in severity level three of the anxiety/depression dimension were comparable to the general population. This could possibly show the emphasis given to experiencing mental health problems in the general population as well, as considerable level of mental health problems are reported in general population samples in Sweden [54, 55].

Following anxiety/depression dimension, while comparable to usual activities dimensions at baseline, pain/discomfort had larger decrements at severity level three mainly among patients from musculoskeletal registers. On the other hand, patients with heart failure, respiratory failure and bipolar disorder assigned large decrements to severity level three of the usual activities dimension. The importance of pain and usual activities dimensions for the respective patient groups seems to go in line with the overall symptoms and the associated implications of the disease/conditions in terms of pain or limitation of day-to-day activities.

At baseline, in most of the patient groups, 20–30% of the variances were explained by EQ-5D-3L dimensions while in the data of respiratory failure (about 14%) and bipolar patients (about 41%) the lowest and the highest proportions were recorded. All were lower than r squared in the model of the general population data (48%). The explained variance increased in all the patient groups at 1-year follow up ranging between 35 and 60% for most patient groups. It showed about 30% explained variance in the model for the patients from the respiratory failure register. The explained variance still remained lower than in the general population for most patient groups with higher proportions in the patients from spine and hip registers and comparable proportions noted among patients from bipolar register. Although not directly comparable, a number of r squared statistics have been reported in regression models of different patient groups including 32% with cervical pre-cancer [34], 39% in those undergoing knee replacement [31] and 47.1% in the eight patients groups cited above [30].

One of the strengths of the present study is the large sample size of patients which allowed investigation of experience-based valuation of health by patients through the EQ VAS and comparison with a large general population sample. The comparison across many patient groups is also an important strength enabling assessment of how specific diseases and associated experience relate to valuation of health states. Furthermore, the study investigated how patients’ valuation of their health changes from baseline to 1-year follow-up. In addition, the study compared OLS models with multilevel models and covariate adjusted models through sensitivity analyses.

On the other hand, an important limitation to take into consideration is possible differences in the way EQ-5D data were collected across the different registers to which some of the difference in valuation could be attributed. In addition, as the state *dead* was not anchored in the present study, its immediate use in economic evaluations could be limited. However, studies among a sample of patients to get their valuations of the state *dead* could remedy this in addition to the current discussion on whether anchoring *dead* is necessary and other alternatives [56]. VAS/ EQ VAS not providing obvious choice or trade-off in the valuation process and the end aversion bias may have had an implication in the EQ VAS valuation in the context of using it in economic valuation [20, 21]. In relation to this, the level of correlation between EQ VAS score and EQ-5D-3L in the different patient groups, even though moderate to high, some level of discrepancy remains between the two measures.

The present study has important implications including showing the feasibility and importance of timing of patient valuations as dimensions important to patients could depend on the type of disease/condition and its stage (e.g., pre- vs. post-operative). This, together with other clinical measures, could facilitate identification of certain aspects of health that may be available for intervention. The broader coverage of EQ VAS than the EQ-5D-3L dimensions was also demonstrated which could emphasize the importance of EQ VAS as a relatively simple but important measure of patients’ overall health. The present study also showed that patient valuations based on EQ VAS scores, elicited through experience-based perspectives, have a potential to be used in the calculation of quality-adjusted life years (QALYs) in comparing different interventions in decision contexts that take patient perspectives into consideration. Furthermore, the study adds information for a discussion on the reconsideration of the need for severity level three in the self-care dimension and to some extent the mobility dimension considering the inconsistencies found in many patient groups. In addition, the findings showed patient valuations could arguably be more appropriate for use in situations where QALYs do not need to be calculated as well; such as summarizing population health survey data and assessing changes in health following surgical and other clinical interventions. The findings also highlighted the importance of mental health among patients with otherwise mainly physical diseases. This provides important information that the mental health aspect is a crucial component in the care of the patients.

The application of clinimetric approaches in future studies of EQ VAS and EQ-5D, besides the current mainly psychometric ones, in assessing patients’ valuations of their health, could provide useful insights in general and in clinical contexts [57, 58].

## Conclusions

The present study showed the consistency between the EQ-5D-3L dimensions and EQ VAS valuations in several patient groups at baseline and at 1-year follow-up. The broader construct which EQ VAS covers, in comparison to the EQ-5D-3L dimensions, was also demonstrated. The main source of inconsistency in terms of decrements was severity level three of the self-care dimension, indicating a possible need to reconsider the importance of this severity level. The study also showed the importance of mental health for overall HRQoL despite the mainly physical nature the conditions of most of the patient groups, with large decrements in the anxiety/depression dimension. Overall, the study revealed crucial contribution of the EQ VAS in the patients’ assessment of their own health, and the potential for these data to provide experience based value sets. The unique advantages of patient value sets in showing aspects of health important to patients in real-world scenarios of valuing health which could be useful inputs for clinical and resource allocation decisions were also demonstrated.

## Supplementary Information

**Additional file 1: Table S1.** Sampling procedure followed including 9 National Quality Registers (NQRs), baseline to 1-year follow-up and the general population data. **Table S2.** Correlation between change in EQ VAS score and change in EQ-5D index across patient groups. **Table S3.** Ordinary least squares regressions in the 9 registers and the general population, baseline, adjusted for sex and age groups. **Table S4.** Mixed model, estimates, baseline. **Table S5.** Mixed model, estimates, baseline, adjusted for sex and age groups. **Table S6.** Ordinary least squares regressions in the 9 registers and the general population, 1-year follow-up, adjusted for sex and age groups. **Table S7.** Mixed model, estimates, 1-year follow-up. **Table S8.** Mixed model, estimates, 1-year follow-up, adjusted for sex and age groups. **Figure S1.** Estimates, OLS models, baseline. **Figure S2.** Estimates, mixed model, baseline. **Figure S3.** Estimates, OLS models, baseline, adjusted for sex and age groups. **Figure S4.** Estimates, mixed model, baseline, adjusted for sex and age groups. **Figure S5.** Estimates, OLS model, 1-year. **Figure S6.** Estimates, mixed model, 1-year. **Figure S7.** Estimates, OLS model, 1-year, adjusted for sex and age groups. **Figure S8.** Estimates, mixed model, 1-year, adjusted for sex and age groups. **Table S9.** Ordinary least squares regressions in the pooled data at baseline. **Table S10.** Ordinary least squares regressions in the pooled data at 1-year follow-up. **Table S11.** Ordinary least squares models of EQ-5D-5L dimensions on EQ VAS score in the BOA and Hip registers.

## Data Availability

Data sharing is not possible according to Swedish law.

## Abbreviations

BOA: Better management of patients with OsteoArthritis
EQ VAS: EuroQol visual analogue scale
EQ-5D: EuroQol five dimensions
EQ-5D-3L: EuroQol 5 dimensions 3 levels
EQ-5D-5L: EuroQol 5 dimensions 5 levels
NQRs: National Quality Registers
OLS: Ordinary least squares regression
QALY: Quality-adjusted life year
SD: Standard deviation
SG: Standard gamble
TTO: Time trade-off
UK: United Kingdom
VAS: Visual analogue scale

## References

[1] Brooks R, Boye KS, Slaap B (2020). EQ-5D: a plea for accurate nomenclature. J Patient Rep Outcomes.

[2] Devlin NJ, Brooks R (2017). EQ-5D and the EuroQol group: past, present and future. Appl Health Econ Health Policy.

[3] Rabin R, de Charro F (2001). EQ-5D: a measure of health status from the EuroQol Group. Ann Med.

[4] EuroQol Research Foundation (2018). EQ-5D-3L User Guide.

[5] EuroQol Research Foundation (2019). EQ-5D-5L User Guide.

[6] Lugnér AK, Krabbe PFM (2020). An overview of the time trade-off method: concept, foundation, and the evaluation of distorting factors in putting a value on health. Expert Rev Pharmacoecon Outcomes Res.

[7] Oppe M, Rand-Hendriksen K, Shah K, Ramos-Goñi JM, Luo N (2016). EuroQol protocols for time trade-off valuation of health outcomes. Pharmacoeconomics.

[8] Burström K, Sun S, Gerdtham U-G, Henriksson M, Johannesson M, Levin L-Å (2014). Swedish experience-based value sets for EQ-5D health states. Qual Life Res.

[9] Burström K, Teni FS, Gerdtham U-G, Leidl R, Helgesson G, Rolfson O (2020). Experience-based Swedish TTO and VAS Value Sets for EQ-5D-5L Health States. Pharmacoeconomics.

[10] Burström K, Johannesson M, Diderichsen F (2006). A comparison of individual and social time trade-off values for health states in the general population. Health Policy.

[11] Lundberg L, Johannesson M, Isacson DGL, Borgquist L (1999). The Relationship between health-state utilities and the sf-12 in a general population. Med Decis Making.

[12] Bardage C, Isacson D, Ring L, Bingefors K (2003). A Swedish population-based study on the relationship between the SF-36 and health utilities to measure health in hypertension. Blood Pressure.

[13] Feng Y, Parkin D, Devlin NJ (2014). Assessing the performance of the EQ-VAS in the NHS PROMs programme. Qual Life Res.

[14] EuroQoL Group (2020). Terminology. EQ-5D.

[15] Johnson JA, Coons SJ, Ergo A, Szava-Kovats G (1998). Valuation of EuroQOL (EQ-5D) health states in an adult US sample. Pharmacoeconomics.

[16] Gutacker N, Patton T, Shah K, Parkin D (2020). Using EQ-5D data to measure hospital performance: are general population values distorting patients’ choices?. Med Decis Making.

[17] Leidl R, Reitmeir P (2011). A value set for the EQ-5D based on experienced health states: development and testing for the German population. Pharmacoeconomics.

[18] Leidl R, Reitmeir P (2017). An Experience-based value set for the EQ-5D-5L in Germany. Value Health.

[19] Sun S, Chen J, Kind P, Xu L, Zhang Y, Burström K (2015). Experience-based VAS values for EQ-5D-3L health states in a national general population health survey in China. Qual Life Res.

[20] Torrance GW, Feeny D, Furlong W (2001). Visual analog scales: do they have a role in the measurement of preferences for health states?. Med Decis Making.

[21] Whitehead SJ, Ali S (2010). Health outcomes in economic evaluation: the QALY and utilities. Br Med Bull.

[22] Neumann PJ, Goldie SJ, Weinstein MC (2000). Preference-Based Measures in Economic Evaluation in Health Care. Annu Rev Public Health.

[23] Brazier J, Green C, McCabe C, Stevens K (2003). Use of visual analog scales in economic evaluation. Expert Rev Pharmacoecon Outcomes Res.

[24] Parkin D, Devlin N (2006). Is there a case for using visual analogue scale valuations in cost-utility analysis?. Health Econ.

[25] Åström M, Lwin ZMT, Teni FS, Burström K, Berg J. Use of the visual analogue scale for health state valuation. Qual Life Res. 2023 (in press). 10.1007/s11136-023-03411-3 PMC10474194 37029258

[26] Brazier J, Rowen D, Karimi M, Peasgood T, Tsuchiya A, Ratcliffe J. Experience-based utility and own health state valuation for a health state classification system: why and how to do it. Eur J Health Econ. 2018;19:881-91. 10.1007/s10198-017-0931-5 PMC6008352 29022120

[27] Cubi-Molla P, Shah K, Burström K (2018). Experience-based values: a framework for classifying different types of experience in health valuation research. Patient.

[28] Nemes S, Burström K, Zethraeus N, Eneqvist T, Garellick G, Rolfson O (2015). Assessment of the Swedish EQ-5D experience-based value sets in a total hip replacement population. Qual Life Res.

[29] Leidl R, Reitmeir P, König H-H, Stark R (2012). The performance of a value set for the EQ-5D based on experienced health states in patients with inflammatory bowel disease. Value Health.

[30] Mann R, Brazier J, Tsuchiya A (2009). A comparison of patient and general population weightings of EQ-5D dimensions. Health Econ.

[31] Pickard AS, Hung Y, Lin F, Lee TA (2017). Patient experience-based value sets: are they stable?. Med Care.

[32] van Dongen JM, van denBerg B, Bekkering GE, van Tulder MW, Ostelo RWJG (2017). Patient versus general population health state valuations: a case study of non-specific low back pain. Qual Life Res.

[33] Whynes DK (2013). Does the correspondence between EQ-5D health state description and VAS score vary by medical condition?. Health Qual Life Outcomes.

[34] Whynes DK, TOMBOLA Group (2008). Correspondence between EQ-5D health state classifications and EQ VAS scores. Health Qual Life Outcomes..

[35] Emilsson L, Lindahl B, Köster M, Lambe M, Ludvigsson JF (2015). Review of 103 Swedish healthcare quality registries. J Intern Med.

[36] Teni FS, Rolfson O, Devlin N, Parkin D, Nauclér E, Burström K (2021). Variations in patients’ overall assessment of their health across and within disease groups using the eq-5d questionnaire: protocol for a longitudinal study in the Swedish national quality registers. JMIR Res Protoc.

[37] Teni FS, Rolfson O, Devlin N, Parkin D, Nauclér E, Burström K (2022). Longitudinal study of patients’ health-related quality of life using EQ-5D-3L in 11 Swedish National Quality Registers. BMJ Open.

[38] Lindgren A, Björk J, Stroh E, Jakobsson K (2010). Adult asthma and traffic exposure at residential address, workplace address, and self-reported daily time outdoor in traffic: a two-stage case-control study. BMC Public Health.

[39] Svensson AC, Fredlund P, Laflamme L, Hallqvist J, Alfredsson L, Ekbom A (2013). Cohort profile: The Stockholm Public Health Cohort. Int J Epidemiol.

[40] Stockholms läns landsting. Hälsoenkät 2006-En undersökning om hälsa och levnadsförhållanden i Stockholms län. 2006 [cited 2021 Aug 22]. Available from: http://dok.slso.sll.se/CES/FHG/Folkhalsoarbete/Halsa%20Stockholm/Enkat-2006unga.pdf

[41] McDonalds JH. Spearman rank correlation. Handbook of Biological Statistics. Baltimore: Sparkey House Publishing; 2014. p. 210–21.

[42] Fowler J, Cohen L, Jarvis P. Measuring correlations. Practical Statistics for Field Biology. 2nd ed. Chichester, West Sussex: Wiley; 2009.

[43] Heijink R, Reitmeir P, Leidl R (2017). International comparison of experience-based health state values at the population level. Health Qual Life Outcomes.

[44] Trudelle-Jackson E, Emerson R, Smith S (2002). Outcomes of total hip arthroplasty: a study of patients one year postsurgery. JOSPT Cases. J Orthop Sports Phys Ther.

[45] García-Gordillo MÁ, del Pozo-Cruz B, Adsuar JC, Cordero-Ferrera JM, Abellán-Perpiñán JM, Sánchez-Martínez FI (2015). Validation and comparison of EQ-5D-3L and SF-6D instruments in a Spanish Parkinson’s disease population sample. Nutr Hosp.

[46] Wu XY, Ohinmaa A, Johnson JA, Veugelers PJ (2014). Assessment of children’s own health status using visual analogue scale and descriptive system of the EQ-5D-Y: linkage between two systems. Qual Life Res.

[47] Åström M, Rolfson O, Burström K. Exploring EQ-5D-Y-3L Experience-based VAS values derived among adolescents. Appl Health Econ Health Policy. [cited 2022 Mar 19]. 10.1007/s40258-021-00713-w 10.1007/s40258-021-00713-w PMC9021108 35083734

[48] Augustovski FA, Irazola VE, Velazquez AP, Gibbons L, Craig BM (2009). Argentine valuation of the EQ-5D health states. Value Health.

[49] Yusof FAM, Goh A, Azmi S (2012). Estimating an EQ-5D value set for Malaysia using time trade-off and visual analogue scale methods. Value Health.

[50] Goudarzi R, Zeraati H, Akbari Sari A, Rashidian A, Mohammad K (2016). Population-based preference weights for the eq-5d health states using the Visual Analogue Scale (VAS) in Iran. Iran Red Crescent Med J.

[51] Rand-Hendriksen K, Augestad LA, Kristiansen IS, Stavem K (2012). Comparison of hypothetical and experienced EQ-5D valuations: relative weights of the five dimensions. Qual Life Res.

[52] Janssen MF, Bonsel GJ, Luo N (2018). Is EQ-5D-5L Better than EQ-5D-3L? A head-to-head comparison of descriptive systems and value sets from seven countries. Pharmacoeconomics.

[53] Law EH, Pickard AS, Xie F, Walton SM, Lee TA, Schwartz A (2018). Parallel valuation: a direct comparison of EQ-5D-3L and EQ-5D-5L societal value sets. Med Decis Making.

[54] Höglund P, Hakelind C, Nordin S (2020). Severity and prevalence of various types of mental ill-health in a general adult population: age and sex differences. BMC Psychiatry.

[55] Olsson S, Hensing G, Burström B, Löve J (2021). Unmet Need for mental healthcare in a population sample in sweden: a cross-sectional study of inequalities based on gender, education, and country of birth. Community Ment Health J.

[56] Sampson C, Parkin D, Devlin N. Drop dead: is anchoring at ‘dead’ a theoretical requirement in health state valuation?. Office of Health Economics; 2020 [cited 2023 Mar 3]. Available from: https://www.ohe.org/publications/drop-dead-anchoring-%E2%80%98dead%E2%80%99-theoretical-requirement-health-state-valuation 10.1002/hec.4863 38831492

[57] Carrozzino D, Patierno C, Guidi J, BerrocalMontiel C, Cao J, Charlson ME (2021). Clinimetric Criteria for Patient-Reported Outcome Measures. Psychother.

[58] Carrozzino D, Patierno C, Pignolo C, Christensen KS. The concept of psychological distress and its assessment: a clinimetric analysis of the SCL-90-R. Int J Stress Manag. 2022. 10.1037/str0000280

