# Supplementary material for: Experience-based health state valuation using the EQ VAS: a register-based study of the EQ-5D-3L among nine patient groups in Sweden
Source: Health Qual Life Outcomes. 2023 Apr 10;21:34. doi: 10.1186/s12955-023-02115-z (PMC10084671; doi:10.1186/s12955-023-02115-z)
Supplement: Supplementary file 1 — Additional file 1: Table S1. Sampling procedure followed including 9 National Quality Registers (NQRs), baseline to 1-year follow-up and the general population data. Table S2. Correlation between change in EQ VAS score and change in EQ-5D index across patient groups. Table S3. Ordinary least squares regressions in the 9 registers and the general population, baseline, adjusted for sex and age groups. Table S4. Mixed model, estimates, baseline. Table S5. Mixed model, estimates, baseline, adjusted for sex and age groups. Table S6. Ordinary least squares regressions in the 9 registers and the general population, 1-year follow-up, adjusted for sex and age groups. Table S7. Mixed model, estimates, 1-year follow-up. Table S8. Mixed model, estimates, 1-year follow-up, adjusted for sex and age groups. Figure S1. Estimates, OLS models, baseline. Figure S2. Estimates, mixed model, baseline. Figure S3. Estimates, OLS models, baseline, adjusted for sex and age groups. Figure S4. Estimates, mixed model, baseline, adjusted for sex and age groups. Figure S5. Estimates, OLS model, 1-year. Figure S6. Estimates, mixed model, 1-year. Figure S7. Estimates, OLS model, 1-year, adjusted for sex and age groups. Figure S8. Estimates, mixed model, 1-year, adjusted for sex and age groups. Table S9. Ordinary least squares regressions in the pooled data at baseline. Table S10. Ordinary least squares regressions in the pooled data at 1-year follow-up. Table S11. Ordinary least squares models of EQ-5D-5L dimensions on EQ VAS score in the BOA and Hip registers. [file 12955_2023_2115_MOESM1_ESM.pdf]

Experience-based health state valuation using the EQ VAS: a register-based study of the EQ-5D-3L among nine patient groups in Sweden

Fitsum Sebsibe Teni[1*], Kristina Burström[1,2], Nancy Devlin[3,4], David Parkin[4,5], Ola Rolfson[1,6,7], The Swedish Quality Register (SWEQR) Study Group

Authors' affiliations

[1] Health Outcomes and Economic Evaluation Research Group, Stockholm Centre for Healthcare Ethics, Department of Learning, Informatics, Management and Ethics, Karolinska Institutet, Stockholm, Sweden.

[2] Equity and Health Policy Research Group, Department of Global Public Health, Karolinska Institutet, Stockholm; Sweden.

[3] Centre for Health Policy, University of Melbourne, Melbourne, Australia.

[4] Office of Health Economics, London, United Kingdom.

[5] City University of London, London, United Kingdom.

[6] Swedish Arthroplasty Register, Gothenburg, Sweden.

[7] Department of Orthopaedics, Institute of Clinical Sciences, Sahlgrenska Academy, University of Gothenburg, Gothenburg, Sweden.

*Corresponding author

Email: fitsum.teni@ki.se

Content

Table S1: Sampling procedure followed including 9 National Quality Registers (NQRs), baseline to 1-year follow-up and the general population data 3

Table S2: Correlation between change in EQ VAS score and change in EQ-5D index across patient groups 4

Table S3: Ordinary least squares regressions in the 9 registers and the general population, baseline, adjusted for sex and age groups 5

Table S4: Mixed model, estimates, baseline 6

Table S5: Mixed model, estimates, baseline, adjusted for sex and age groups 7

Table S6: Ordinary least squares regressions in the 9 registers and the general population, 1-year follow-up, adjusted for sex and age groups 8

Table S7: Mixed model, estimates, 1-year follow-up 9

Table S8: Mixed model, estimates, 1-year follow-up, adjusted for sex and age groups 10

Figure S1: Estimates, OLS models, baseline 11

Figure S2: Estimates, mixed model, baseline 12

Figure S3: Estimates, OLS models, baseline, adjusted for sex and age groups 13

Figure S4: Estimates, mixed model, baseline, adjusted for sex and age groups 14

Figure S5: Estimates, OLS model, 1-year 15

Figure S6: Estimates, mixed model, 1-year 16

Figure S7: Estimates, OLS model, 1-year, adjusted for sex and age groups 17

Figure S8: Estimates, mixed model, 1-year, adjusted for sex and age groups 18

Table S9: Ordinary least squares regressions in the pooled data at baseline 19

Table S10: Ordinary least squares regressions in the pooled data at 1-year follow-up 20

Table S11: Ordinary least squares models of EQ-5D-5L dimensions on EQ VAS score in the BOA and Hip registers 21

Table S1: Sampling procedure followed including 9 National Quality Registers (NQRs), baseline to 1-year follow-up and the general population data

Category	Patient groups in NQRs	A	B	C	D	
		n	n	n	n	%
Intervention-based registers	Spine	53,418	53,015	51,047	44,196	20.7
	Hip	95,887	93,684	90,669	90,658	42.4
	Knee	17,600	16,324	16,324	16,324	7.6
	Ankle	794	789	725	668	0.3
	Cruciate ligament	10,532	10,532	10,465	8,155	3.8
	Osteoarthritis (BOA)	15,238	14,877	13,965	6,690	3.1
Diagnosis-based registers	Heart failure	1,789	1,746	1,691	1,044	0.5
	Respiratory failure	1,287	1,063	1,050	725	0.3
	Bipolar	3,947	3,946	3,823	3,610	1.7
NQRs (total, count)		200,492	195,976	189,759	172,070	-
General population data		49,499	49,999	49,999	41,761	19.5
**Total**		**249,991**	**245,475**	**239,258**	**213,831**	**100.0**

A= Data on baseline and 1-year follow-up
B= After selection of data with information on diagnosis and intervention
C= After de-duplication of records occurring in more than one patient group (duplicate records taken out keeping one record)
D= Included in the analyses of EQ VAS values
BOA=Better management of OsteoArthritis register, referred to as BOA in whole manuscript

Table S2: Correlation between change in EQ VAS score and change in EQ-5D index across patient groups

Register/ general population		Spearman's correlation	
		Change	
		Coefficient	P-value
**Intervention-based**	**Spine**	0.573	<0.0001
	**Hip**	0.452	<0.0001
	**Knee**	0.392	<0.0001
	**Ankle**	0.478	<0.0001
	**Cruciate ligament**	0.373	<0.0001
	**BOA**	0.339	<0.0001
**Diagnosis-based**	**Heart failure**	0.300	<0.0001
	**Respiratory failure**	0.294	<0.0001
	**Bipolar**	0.386	<0.0001

BOA: Better management of OsteoArthritis

Table S3: Ordinary least squares regressions in the 9 registers and the general population, baseline, adjusted for sex and age groups

Patient group	Spine			Hip			Knee			Ankle			Cruciate		
**EQ-5D-3L**	**Estimate**	**RSE**	**P-value**	**Estimate**	**RSE**	**P-value**	**Estimate**	**RSE**	**P-value**	**Estimate**	**RSE**	**P-value**	**Estimate**	**RSE**	**P-value**
Intercept	78.28	1.37	<.0001	75.23	2.09	<.0001	79.06	1.81	<.0001	84.8	6.7	<.0001	78.90	0.61	<.0001
MO_2	-5.92	0.25	<.0001	-5.81	0.24	<.0001	-6.92	0.45	<.0001	-2.8	4.8	0.5547	-7.70	0.56	<.0001
MO_3	-12.04	0.84	<.0001	-10.56	1.36	<.0001	-4.67	3.82	0.2211	-6.4	7.2	0.3722	-9.58	4.21	0.0228
SC_2	-3.89	0.25	<.0001	-4.03	0.17	<.0001	-5.63	0.76	<.0001	-5.3	2.7	0.0521	-7.09	1.72	<.0001
SC_3	-3.40	0.96	0.0004	-2.93	0.75	<.0001	-0.62	1.80	0.7309	1.9	8.5	0.8210	-0.29	3.05	0.9247
UA_2	-6.36	0.22	<.0001	-5.68	0.15	<.0001	-6.79	0.35	<.0001	-6.4	1.7	0.0002	-3.17	0.52	<.0001
UA_3	-12.99	0.31	<.0001	-12.14	0.28	<.0001	-11.29	0.88	<.0001	-14.2	2.9	<.0001	-7.00	0.92	<.0001
PD_2	-5.77	1.31	<.0001	-2.24	0.58	0.0001	-2.71	1.05	0.0095	-9.1	2.4	0.0002	-5.11	0.65	<.0001
PD_3	-16.18	1.32	<.0001	-10.86	0.59	<.0001	-9.54	1.10	<.0001	-19.3	2.6	<.0001	-13.99	1.32	<.0001
AD_2	-6.77	0.19	<.0001	-7.57	0.14	<.0001	-8.72	0.35	<.0001	-5.5	1.7	0.0010	-9.75	0.49	<.0001
AD_3	-14.26	0.42	<.0001	-16.37	0.41	<.0001	-18.08	1.21	<.0001	-15.4	3.8	<.0001	-22.00	1.14	<.0001
Adjusted R^2	0.2844			0.2223			0.2076			0.2417			0.2079		
RMSE	18.69			19.54			19.66			18.68			20.48		
**Patient group**	**BOA**			**Heart failure**			**Respiratory failure**			**Bipolar**			**General population**		
**EQ-5D-3L**	**Estimate**	**RSE**	**P-value**	**Estimate**	**RSE**	**P-value**	**Estimate**	**RSE**	**P-value**	**Estimate**	**RSE**	**P-value**	**Estimate**	**RSE**	**P-value**
Intercept	97.53	4.22	<.0001	61.13	8.21	<.0001	65.90	2.78	<.0001	80.81	0.85	<.0001	88.40	0.17	<.0001
MO_2	-6.78	0.42	<.0001	-5.30	1.28	<.0001	-4.23	2.08	0.0423	-3.79	0.92	<.0001	-9.77	0.37	<.0001
MO_3	-11.38	3.75	0.0024	7.00	3.40	0.0394	1.81	6.79	0.7902	-0.29	8.83	0.9734	-2.43	5.07	0.6315
SC_2	-3.55	1.17	0.0025	6.51	2.38	0.0063	-1.41	1.77	0.4269	-4.05	1.43	0.0045	-3.63	0.97	0.0002
SC_3	3.07	3.23	0.3408	9.09	8.86	0.3055	-2.02	3.38	0.5518	-4.48	6.70	0.5032	0.71	1.69	0.6761
UA_2	-7.28	0.52	<.0001	-9.41	1.36	<.0001	-5.23	2.01	0.0094	-8.17	0.75	<.0001	-12.41	0.41	<.0001
UA_3	-15.25	1.90	<.0001	-24.61	3.11	<.0001	-11.74	2.66	<.0001	-14.34	1.80	<.0001	-18.06	1.00	<.0001
PD_2	-6.92	1.23	<.0001	-2.80	1.12	0.013	-1.87	1.83	0.3060	-2.49	0.61	<.0001	-6.78	0.14	<.0001
PD_3	-15.26	1.48	<.0001	-5.34	2.58	0.0386	-7.83	2.70	0.0038	-6.38	1.30	<.0001	-20.13	0.59	<.0001
AD_2	-8.62	0.46	<.0001	-8.78	1.12	<.0001	-4.11	1.59	0.0097	-13.98	0.60	<.0001	-10.13	0.17	<.0001
AD_3	-18.94	2.18	<.0001	-18.14	3.66	<.0001	-12.62	3.26	0.0001	-30.96	1.23	<.0001	-30.65	0.71	<.0001
Adjusted R^2	0.2689			0.2854			0.1380			0.4091			0.4859		
RMSE	15.88			15.98			19.76			16.18			13.10		

BOA: Better management of OsteoArthritis; RMSE: Root mean square error; RSE: Robust standard error; Darker shades under estimate columns show inconsistency in decrement; Lighter shades in the P-value columns show non-statistically significant estimates

Table S4: Mixed model, estimates, baseline

Patient group	Spine			Hip			Knee			Ankle			Cruciate ligament		
EQ-5D-3L	Estimate	SE	P-value	Estimate	SE	P-value	Estimate	SE	P-value	Estimate	SE	P-value	Estimate	SE	P-value
MO_2	-5.84	0.25	<.0001	-5.86	0.23	<.0001	-6.91	0.46	<.0001	-4.95	2.29	0.0458	-7.94	0.48	<.0001
MO_3	-11.55	0.71	<.0001	-10.35	1.06	<.0001	-7.74	2.08	0.0009	-7.92	2.79	0.0177	-9.15	2.34	0.0014
SC_2	-3.73	0.24	<.0001	-3.84	0.16	<.0001	-5.35	0.63	<.0001	-4.49	1.70	0.0209	-5.65	1.28	<.0001
SC_3	-2.91	0.81	0.0005	-2.68	0.62	<.0001	-1.43	1.31	0.2823	-1.51	2.02	0.4813	-1.67	1.76	0.3625
UA_2	-6.58	0.22	<.0001	-5.67	0.14	<.0001	-6.84	0.32	<.0001	-6.50	1.40	<.0001	-3.21	0.45	<.0001
UA_3	-13.11	0.29	<.0001	-11.89	0.24	<.0001	-11.24	0.67	<.0001	-13.14	1.84	<.0001	-7.34	0.75	<.0001
PD_2	-5.66	1.06	<.0001	-2.30	0.50	<.0001	-2.98	1.04	0.0044	-3.85	2.72	0.1729	-5.39	0.58	<.0001
PD_3	-16.33	1.07	<.0001	-11.38	0.51	<.0001	-10.32	1.07	<.0001	-13.93	2.73	<.0001	-14.43	1.04	<.0001
AD_2	-6.94	0.19	<.0001	-7.99	0.13	<.0001	-9.17	0.32	<.0001	-6.38	1.48	<.0001	-9.49	0.42	<.0001
AD_3	-14.50	0.39	<.0001	-17.04	0.35	<.0001	-19.07	0.94	<.0001	-18.16	2.84	<.0001	-21.57	0.92	<.0001
**Patient group**	**BOA**			**Heart failure**			**Respiratory failure**			**Bipolar**			**General population**		
**EQ-5D-3L**	**Estimate**	**SE**	**P-value**	**Estimate**	**SE**	**P-value**	**Estimate**	**SE**	**P-value**	**Estimate**	**SE**	**P-value**	**Estimate**	**SE**	**P-value**
MO_2	-6.66	0.48	<.0001	-5.66	1.27	<.0001	-4.75	1.58	0.0036	-3.22	0.94	0.0006	-9.89	0.38	<.0001
MO_3	-9.33	2.53	0.0044	-7.62	2.64	0.0173	-5.97	2.61	0.0409	-6.30	2.52	0.0295	-7.29	2.08	0.0018
SC_2	-3.61	1.13	0.0023	-0.20	1.69	0.9077	-1.83	1.41	0.2076	-3.85	1.34	0.0073	-3.64	0.88	<.0001
SC_3	-0.81	1.83	0.6697	0.67	2.02	0.7500	-0.09	1.89	0.9623	-1.36	1.92	0.5028	0.01	1.33	0.9913
UA_2	-7.37	0.57	<.0001	-7.73	1.37	<.0001	-5.48	1.49	0.0003	-8.32	0.75	<.0001	-12.23	0.40	<.0001
UA_3	-14.26	1.56	<.0001	-15.72	2.04	<.0001	-11.50	1.83	<.0001	-14.21	1.46	<.0001	-17.45	0.91	<.0001
PD_2	-6.05	1.34	<.0001	-3.19	1.20	0.0082	-2.15	1.49	0.149	-2.44	0.66	0.0002	-6.73	0.19	<.0001
PD_3	-14.42	1.50	<.0001	-9.30	2.15	<.0001	-8.86	2.00	<.0001	-7.49	1.26	<.0001	-19.88	0.56	<.0001
AD_2	-8.95	0.50	<.0001	-8.18	1.23	<.0001	-4.48	1.41	0.0015	-14.03	0.68	<.0001	-10.06	0.21	<.0001
AD_3	-19.96	1.88	<.0001	-18.17	2.69	<.0001	-13.49	2.31	<.0001	-30.68	1.12	<.0001	-30.40	0.59	<.0001

BOA: Better management of OsteoArthritis; RMSE: Root mean square error; RSE: Robust standard error; Darker shades under estimate columns show inconsistency in decrement; Lighter shades in the P-value columns show non-statistically significant estimates

Table S5: Mixed model, estimates, baseline, adjusted for sex and age groups

Patient group	Spine			Hip			Knee			Ankle			Cruciate ligament		
**EQ-5D-3L**	**Estimate**	**SE**	**P-value**	**Estimate**	**SE**	**P-value**	**Estimate**	**SE**	**P-value**	**Estimate**	**SE**	**P-value**	**Estimate**	**SE**	**P-value**
MO_2	-5.92	0.26	<.0001	-5.79	0.23	<.0001	-6.84	0.46	<.0001	-5.03	2.27	0.0425	-7.70	0.48	<.0001
MO_3	-11.75	0.71	<.0001	-10.09	1.06	<.0001	-7.65	2.06	0.0011	-7.98	2.76	0.0168	-8.99	2.32	0.0016
SC_2	-3.89	0.24	<.0001	-4.03	0.16	<.0001	-5.41	0.62	<.0001	-4.48	1.73	0.0211	-5.65	1.28	<.0001
SC_3	-3.00	0.81	0.0003	-2.76	0.62	<.0001	-1.36	1.31	0.3051	-1.40	2.08	0.5246	-1.51	1.79	0.4135
UA_2	-6.37	0.22	<.0001	-5.69	0.14	<.0001	-6.84	0.32	<.0001	-6.45	1.39	<.0001	-3.33	0.45	<.0001
UA_3	-13.02	0.29	<.0001	-12.16	0.24	<.0001	-11.51	0.67	<.0001	-13.39	1.83	<.0001	-7.61	0.75	<.0001
PD_2	-5.51	1.06	<.0001	-2.30	0.50	<.0001	-2.85	1.04	0.0064	-3.83	2.75	0.1790	-5.06	0.59	<.0001
PD_3	-15.92	1.07	<.0001	-10.93	0.51	<.0001	-9.73	1.07	<.0001	-13.63	2.76	<.0001	-13.90	1.04	<.0001
AD_2	-6.76	0.19	<.0001	-7.58	0.13	<.0001	-8.73	0.32	<.0001	-6.02	1.49	<.0001	-9.67	0.43	<.0001
AD_3	-14.31	0.39	<.0001	-16.41	0.35	<.0001	-18.34	0.94	<.0001	-17.50	2.87	<.0001	-21.82	0.92	<.0001
**Patient group**	**BOA**			**Heart failure**			**Respiratory failure**			**Bipolar**			**General population**		
**EQ-5D-3L**	**Estimate**	**SE**	**P-value**	**Estimate**	**SE**	**P-value**	**Estimate**	**SE**	**P-value**	**Estimate**	**SE**	**P-value**	**Estimate**	**SE**	**P-value**
MO_2	-6.80	0.48	<.0001	-5.51	1.28	<.0001	-4.76	1.57	0.0035	-3.52	0.95	0.0002	-9.84	0.38	<.0001
MO_3	-9.33	2.50	0.0044	-7.56	2.61	0.0178	-6.00	2.58	0.0393	-6.50	2.50	0.0252	-7.23	2.07	0.0019
SC_2	-3.67	1.13	0.0018	0.04	1.72	0.9800	-1.74	1.42	0.2308	-3.86	1.35	0.0068	-3.57	0.88	<.0001
SC_3	-0.69	1.87	0.7218	0.89	2.08	0.6835	0.05	1.93	0.9779	-1.31	1.97	0.5265	0.18	1.34	0.8939
UA_2	-7.31	0.57	<.0001	-7.72	1.36	<.0001	-5.57	1.48	0.0003	-8.24	0.75	<.0001	-12.32	0.40	<.0001
UA_3	-14.42	1.55	<.0001	-15.81	2.02	<.0001	-11.63	1.82	<.0001	-14.23	1.45	<.0001	-17.50	0.91	<.0001
PD_2	-6.09	1.34	<.0001	-3.12	1.20	0.0096	-2.13	1.49	0.1531	-2.48	0.67	0.0002	-6.79	0.20	<.0001
PD_3	-14.35	1.50	<.0001	-9.03	2.16	<.0001	-8.74	2.00	<.0001	-7.38	1.27	<.0001	-20.01	0.56	<.0001
AD_2	-8.66	0.50	<.0001	-8.21	1.24	<.0001	-4.55	1.42	0.0013	-13.92	0.68	<.0001	-10.14	0.21	<.0001
AD_3	-19.65	1.88	<.0001	-18.32	2.70	<.0001	-13.66	2.33	<.0001	-30.50	1.12	<.0001	-30.51	0.59	<.0001

BOA: Better management of OsteoArthritis; RMSE: Root mean square error; RSE: Robust standard error; Darker shades under estimate columns show inconsistency in decrement; Lighter shades in the P-value columns show non-statistically significant estimates

Table S6: Ordinary least squares regressions in the 9 registers and the general population, 1-year follow-up, adjusted for sex and age groups

Patient group	Spine			Hip			Knee			Ankle			Cruciate		
EQ-5D-3L	Estimate	RSE	P-value	Estimate	RSE	P-value	Estimate	RSE	P-value	Estimate	RSE	P-value	Estimate	RSE	P-value
Intercept	90.38	0.34	<.0001	90.97	1.16	<.0001	90.50	1.78	<.0001	85.54	4.05	<.0001	86.50	0.34	<.0001
MO_2	-9.29	0.19	<.0001	-8.46	0.14	<.0001	-8.56	0.31	<.0001	-7.88	1.32	<.0001	-8.83	0.74	<.0001
MO_3	-10.77	1.93	<.0001	-11.35	2.22	<.0001	-14.20	4.28	0.0009	-27.81	6.87	<.0001	-3.32	12.61	0.7921
SC_2	-3.43	0.34	<.0001	-3.24	0.24	<.0001	-6.08	0.79	<.0001	-6.90	2.51	0.0062	-4.41	2.43	0.0694
SC_3	-1.91	1.10	0.0819	-4.67	0.84	<.0001	-6.08	2.22	0.0062	6.30	4.01	0.1165	2.81	3.16	0.3742
UA_2	-8.93	0.21	<.0001	-8.54	0.16	<.0001	-6.43	0.38	<.0001	-7.20	1.58	<.0001	-5.41	0.55	<.0001
UA_3	-16.55	0.47	<.0001	-17.23	0.50	<.0001	-14.73	1.33	<.0001	-11.04	3.54	0.0019	-9.27	1.88	<.0001
PD_2	-9.48	0.16	<.0001	-7.88	0.11	<.0001	-6.67	0.25	<.0001	-2.24	1.36	0.0984	-6.58	0.40	<.0001
PD_3	-23.10	0.34	<.0001	-22.43	0.36	<.0001	-18.34	0.84	<.0001	-15.40	3.03	<.0001	-19.28	1.50	<.0001
AD_2	-9.07	0.18	<.0001	-9.86	0.15	<.0001	-10.21	0.36	<.0001	-6.45	1.50	<.0001	-10.00	0.45	<.0001
AD_3	-19.11	0.49	<.0001	-23.02	0.61	<.0001	-22.85	1.46	<.0001	-21.00	4.34	<.0001	-25.69	1.39	<.0001
Adjusted R^2	0.5977			0.5239			0.4492			0.4581			0.3533		
RMSE	14.27			13.83			14.41			13.88			15.99		

Patient group	BOA			Heart failure			Respiratory failure			Bipolar			General population		
EQ-5D-3L	Estimate	RSE	P-value	Estimate	RSE	P-value	Estimate	RSE	P-value	Estimate	RSE	P-value	Estimate	RSE	P-value
Intercept	92.46	15.62	<.0001	77.82	4.39	<.0001	73.57	2.88	<.0001	82.16	0.75	<.0001	88.40	0.17	<.0001
MO_2	-9.62	0.41	<.0001	-5.02	1.17	<.0001	-8.11	1.81	<.0001	-3.02	0.80	0.0002	-9.77	0.37	<.0001
MO_3	-11.81	6.02	0.0497	12.50	7.68	0.1037	-11.51	4.17	0.0059	0.82	5.23	0.8756	-2.43	5.07	0.6315
SC_2	-4.29	1.28	0.0008	-6.92	2.27	0.0023	-4.93	1.40	0.0004	-2.95	1.30	0.0234	-3.63	0.97	0.0002
SC_3	-6.31	2.47	0.0106	-11.66	9.25	0.2078	-9.07	3.67	0.0137	-6.23	6.55	0.3410	0.71	1.69	0.6761
UA_2	-7.95	0.57	<.0001	-6.74	1.35	<.0001	-4.87	1.83	0.0080	-9.57	0.70	<.0001	-12.41	0.41	<.0001
UA_3	-13.54	3.09	<.0001	-13.04	4.13	0.0016	-9.48	2.20	<.0001	-14.45	1.80	<.0001	-18.06	1.00	<.0001
PD_2	-9.41	0.52	<.0001	-3.79	1.04	0.0003	-5.11	1.72	0.0031	-2.57	0.56	<.0001	-6.78	0.14	<.0001
PD_3	-20.01	1.06	<.0001	-6.73	2.25	0.0028	-8.70	2.27	0.0001	-10.86	1.19	<.0001	-20.13	0.59	<.0001
AD_2	-9.51	0.44	<.0001	-9.97	1.12	<.0001	-5.53	1.45	0.0001	-12.71	0.54	<.0001	-10.13	0.17	<.0001
AD_3	-20.31	2.20	<.0001	-24.83	3.41	<.0001	-14.19	2.40	<.0001	-30.12	1.09	<.0001	-30.65	0.71	<.0001
Adjusted R^2	0.4263			0.3653			0.2931			0.4731			0.4859		
RMSE	14.17			14.97			17.14			14.62			13.10		

BOA: Better management of OsteoArthritis; RMSE: Root mean square error; RSE: Robust standard error; Darker shades under estimate columns show inconsistency in decrement; Lighter shades in the P-value columns show non-statistically significant estimates

Table S7: Mixed model, estimates, 1-year follow-up

Patient group	Spine			Hip			Knee			Ankle			Cruciate ligament		
EQ-5D-3L	Estimate	SE	P-value	Estimate	SE	P-value	Estimate	SE	P-value	Estimate	SE	P-value	Estimate	SE	P-value
MO_2	-9.94	0.17	<.0001	-8.84	0.13	<.0001	-8.73	0.28	<.0001	-7.67	1.34	<.0001	-8.96	0.54	<.0001
MO_3	-11.04	1.25	<.0001	-11.48	1.20	<.0001	-11.77	2.04	<.0001	-10.35	2.72	0.0019	-8.82	2.56	0.0035
SC_2	-3.41	0.27	<.0001	-3.42	0.20	<.0001	-6.06	0.56	<.0001	-5.19	1.51	0.0031	-3.87	1.35	0.0081
SC_3	-2.44	0.86	0.005	-4.72	0.61	<.0001	-5.54	1.21	<.0001	-3.51	1.78	0.0774	-2.14	1.60	0.1991
UA_2	-8.78	0.18	<.0001	-8.49	0.14	<.0001	-6.43	0.32	<.0001	-7.44	1.21	<.0001	-5.54	0.44	<.0001
UA_3	-16.31	0.38	<.0001	-17.16	0.37	<.0001	-14.75	0.87	<.0001	-13.63	1.79	<.0001	-10.78	1.13	<.0001
PD_2	-9.45	0.18	<.0001	-7.79	0.12	<.0001	-6.58	0.26	<.0001	-2.19	1.44	0.1292	-6.67	0.36	<.0001
PD_3	-22.98	0.29	<.0001	-22.13	0.27	<.0001	-18.19	0.59	<.0001	-15.30	2.16	<.0001	-19.22	1.03	<.0001
AD_2	-8.96	0.16	<.0001	-9.85	0.13	<.0001	-10.23	0.30	<.0001	-6.96	1.32	<.0001	-9.69	0.37	<.0001
AD_3	-18.87	0.37	<.0001	-22.88	0.40	<.0001	-22.98	0.88	<.0001	-22.15	2.51	<.0001	-24.93	0.93	<.0001

Patient group	BOA			Heart failure			Respiratory failure			Bipolar			General population		
EQ-5D-3L	Estimate	SE	P-value	Estimate	SE	P-value	Estimate	SE	P-value	Estimate	SE	P-value	Estimate	SE	P-value
MO_2	-9.80	0.39	<.0001	-6.02	1.02	<.0001	-7.63	1.39	<.0001	-2.82	0.70	<.0001	-9.89	0.29	<.0001
MO_3	-10.43	2.57	0.001	-5.70	2.64	0.0469	-9.72	2.32	0.0002	-4.22	2.30	0.0774	-5.59	1.83	0.0032
SC_2	-4.41	0.92	<.0001	-5.61	1.36	0.0003	-4.12	1.09	0.0003	-3.00	0.99	0.0032	-3.62	0.67	<.0001
SC_3	-4.40	1.55	0.0123	-4.24	1.76	0.0370	-4.82	1.54	0.0052	-2.97	1.67	0.1016	-0.67	1.05	0.5274
UA_2	-7.96	0.49	<.0001	-7.00	1.06	<.0001	-6.22	1.25	<.0001	-9.47	0.59	<.0001	-12.21	0.31	<.0001
UA_3	-14.28	1.45	<.0001	-13.70	1.72	<.0001	-12.02	1.45	<.0001	-14.60	1.15	<.0001	-17.58	0.71	<.0001
PD_2	-9.28	0.59	<.0001	-3.62	0.99	0.0002	-5.27	1.27	<.0001	-2.48	0.52	<.0001	-6.74	0.15	<.0001
PD_3	-19.70	0.91	<.0001	-8.01	1.69	<.0001	-9.47	1.70	<.0001	-11.05	0.97	<.0001	-20.04	0.44	<.0001
AD_2	-9.51	0.41	<.0001	-9.48	0.96	<.0001	-6.06	1.11	<.0001	-12.69	0.52	<.0001	-10.05	0.16	<.0001
AD_3	-21.08	1.54	<.0001	-23.29	2.14	<.0001	-15.87	1.76	<.0001	-29.81	0.87	<.0001	-30.40	0.45	<.0001

BOA: Better management of OsteoArthritis; RMSE: Root mean square error; RSE: Robust standard error; Darker shades under estimate columns show inconsistency in decrement; Lighter shades in the P-value columns show non-statistically significant estimates

Table S8: Mixed model, estimates, 1-year follow-up, adjusted for sex and age groups

Patient group	Spine			Hip			Knee			Ankle			Cruciate ligament		
EQ-5D-3L	Estimate	SE	P-value	Estimate	SE	P-value	Estimate	SE	P-value	Estimate	SE	P-value	Estimate	SE	P-value
MO_2	-9.32	0.18	<.0001	-8.47	0.13	<.0001	-8.53	0.29	<.0001	-7.55	1.34	<.0001	-8.78	0.54	<.0001
MO_3	-10.45	1.25	<.0001	-10.95	1.20	<.0001	-11.55	2.03	<.0001	-10.08	2.71	0.0022	-8.41	2.55	0.0046
SC_2	-3.45	0.27	<.0001	-3.27	0.20	<.0001	-5.92	0.56	<.0001	-5.00	1.49	0.0038	-3.75	1.33	0.0095
SC_3	-2.27	0.85	0.0085	-4.46	0.61	<.0001	-5.26	1.20	<.0001	-3.27	1.74	0.0897	-1.96	1.57	0.2300
UA_2	-8.92	0.18	<.0001	-8.54	0.14	<.0001	-6.51	0.33	<.0001	-7.51	1.21	<.0001	-5.55	0.44	<.0001
UA_3	-16.47	0.38	<.0001	-17.18	0.37	<.0001	-14.81	0.87	<.0001	-13.66	1.80	<.0001	-10.72	1.13	<.0001
PD_2	-9.47	0.18	<.0001	-7.88	0.12	<.0001	-6.67	0.26	<.0001	-2.39	1.44	0.0958	-6.57	0.36	<.0001
PD_3	-23.08	0.29	<.0001	-22.41	0.27	<.0001	-18.35	0.59	<.0001	-15.49	2.15	<.0001	-19.05	1.03	<.0001
AD_2	-9.07	0.16	<.0001	-9.86	0.13	<.0001	-10.22	0.30	<.0001	-6.99	1.31	<.0001	-9.94	0.38	<.0001
AD_3	-19.17	0.37	<.0001	-23.06	0.40	<.0001	-23.05	0.88	<.0001	-22.35	2.49	<.0001	-25.31	0.93	<.0001
Patient group	BOA			Heart failure			Respiratory failure			Bipolar			General population		
EQ-5D-3L	Estimate	SE	P-value	Estimate	SE	P-value	Estimate	SE	P-value	Estimate	SE	P-value	Estimate	SE	P-value
MO_2	-9.60	0.39	<.0001	-5.46	1.02	<.0001	-7.51	1.36	<.0001	-2.65	0.70	0.0002	-9.79	0.29	<.0001
MO_3	-10.19	2.55	0.0012	-5.41	2.62	0.0563	-9.51	1.88	0.0002	-4.01	2.29	0.0908	-5.30	1.82	0.0049
SC_2	-4.35	0.92	<.0001	-5.34	1.34	0.0005	-4.03	1.11	0.0004	-2.88	0.99	0.0044	-3.51	0.67	<.0001
SC_3	-4.15	1.52	0.0154	-3.95	1.72	0.0451	-4.52	1.59	0.0075	-2.77	1.63	0.1168	-0.55	1.04	0.5987
UA_2	-7.99	0.49	<.0001	-7.02	1.06	<.0001	-6.29	1.28	<.0001	-9.55	0.59	<.0001	-12.32	0.31	<.0001
UA_3	-14.27	1.46	<.0001	-13.66	1.72	<.0001	-12.07	1.48	<.0001	-14.62	1.15	<.0001	-17.62	0.71	<.0001
PD_2	-9.33	0.59	<.0001	-3.74	0.99	0.0001	-5.25	1.27	<.0001	-2.52	0.52	<.0001	-6.78	0.15	<.0001
PD_3	-19.82	0.91	<.0001	-8.18	1.69	<.0001	-9.49	1.70	<.0001	-11.17	0.97	<.0001	-20.14	0.44	<.0001
AD_2	-9.53	0.41	<.0001	-9.63	0.96	<.0001	-6.20	1.11	<.0001	-12.75	0.52	<.0001	-10.14	0.16	<.0001
AD_3	-21.22	1.53	<.0001	-23.73	2.12	<.0001	-16.29	1.74	<.0001	-29.88	0.87	<.0001	-30.53	0.45	<.0001

BOA: Better management of OsteoArthritis; RMSE: Root mean square error; RSE: Robust standard error; Darker shades under estimate columns show inconsistency in decrement; Lighter shades in the P-value columns show non-statistically significant estimates

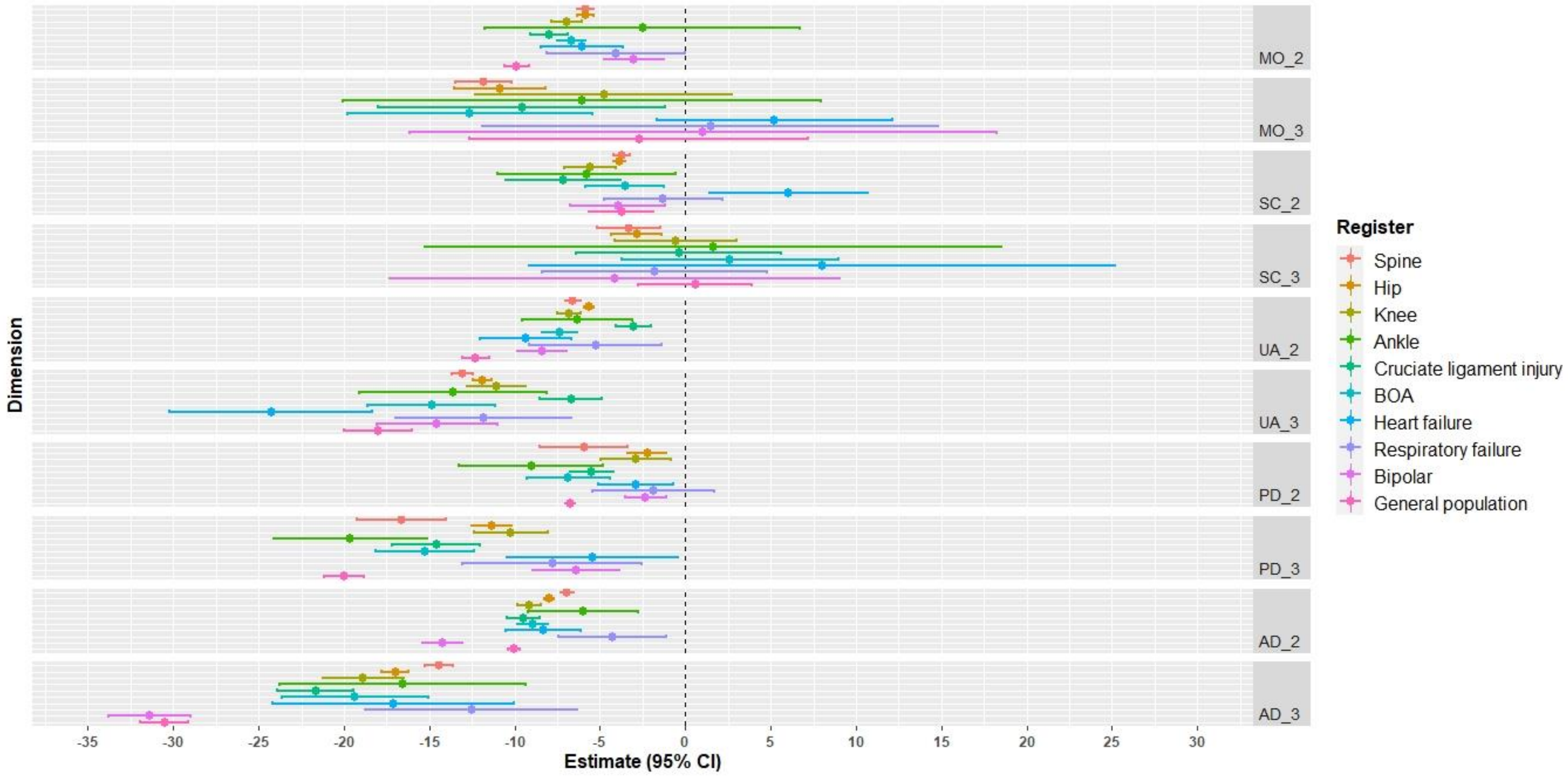

Figure S1: Estimates, OLS models, baseline

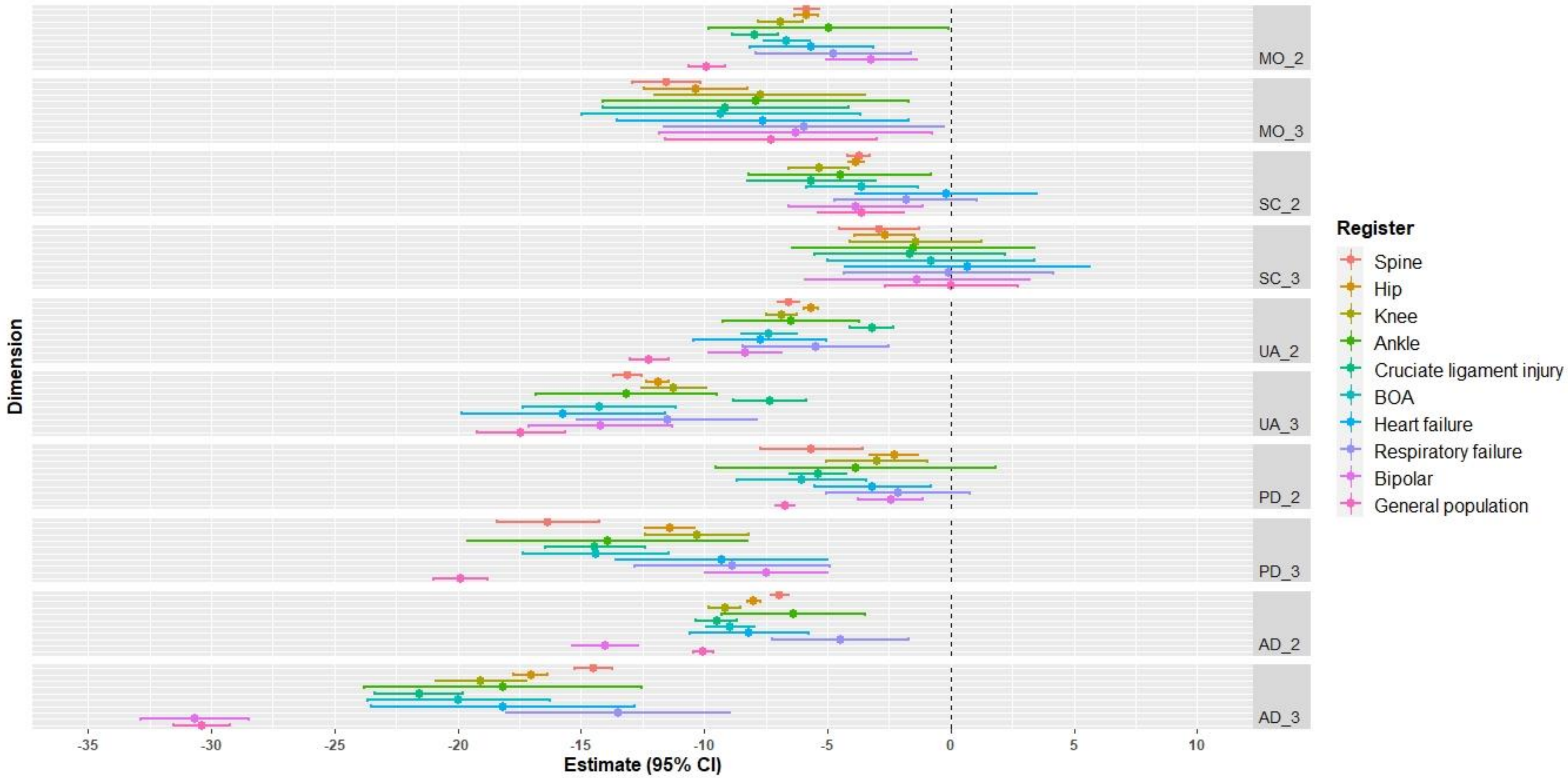

Figure S2: Estimates, mixed model, baseline

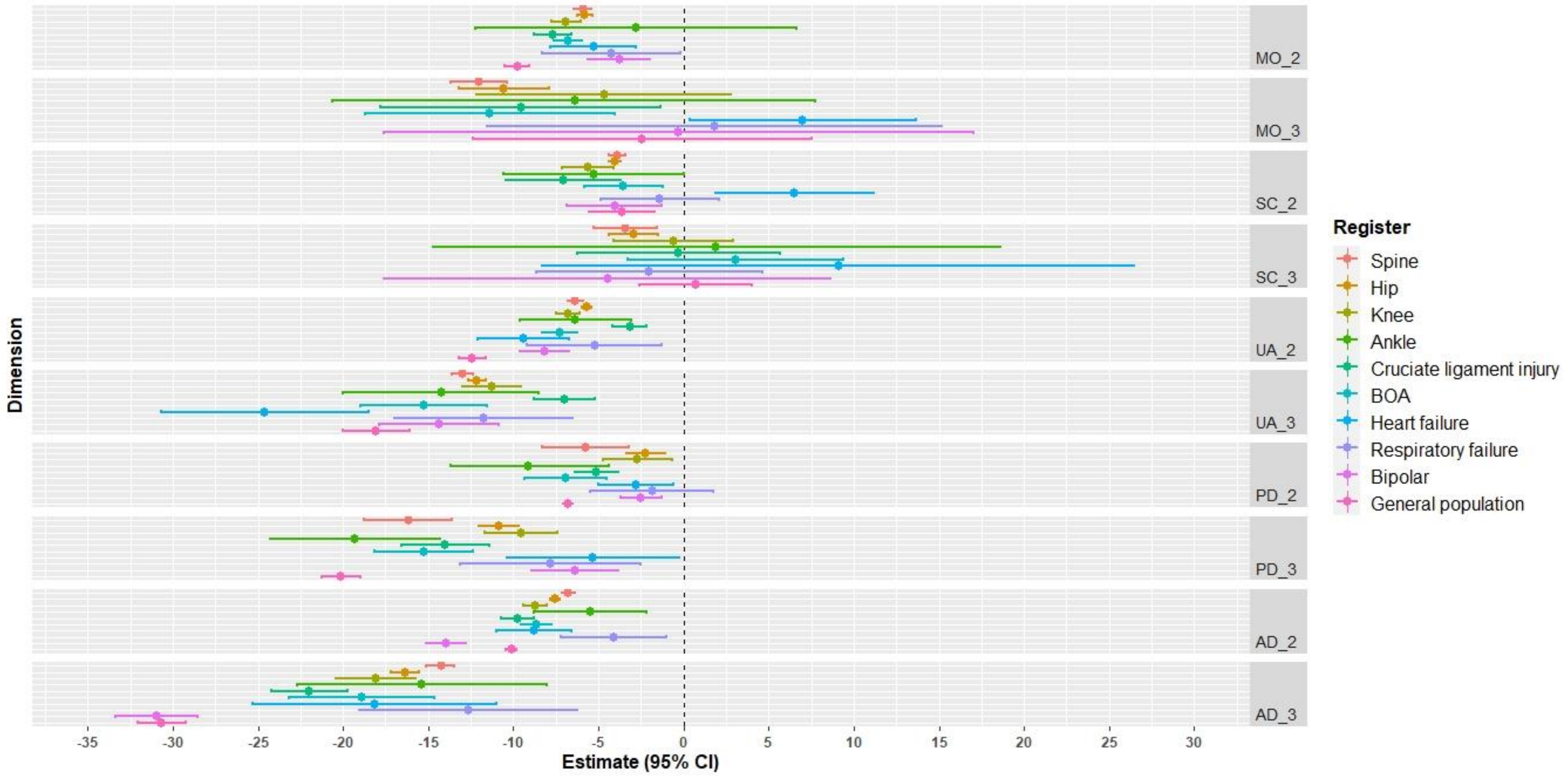

Figure S3: Estimates, OLS models, baseline, adjusted for sex and age groups

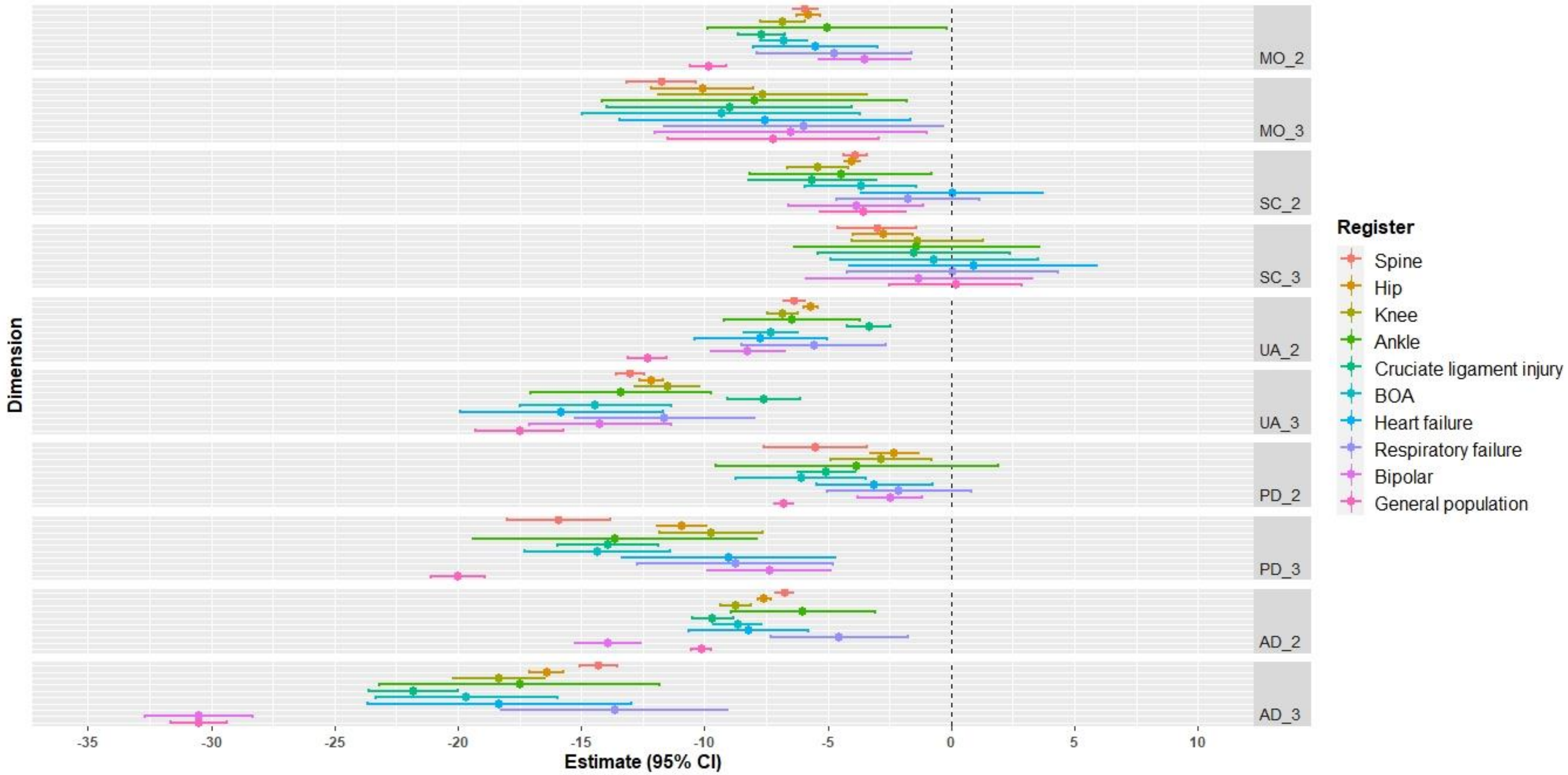

Figure S4: Estimates, mixed model, baseline, adjusted for sex and age groups

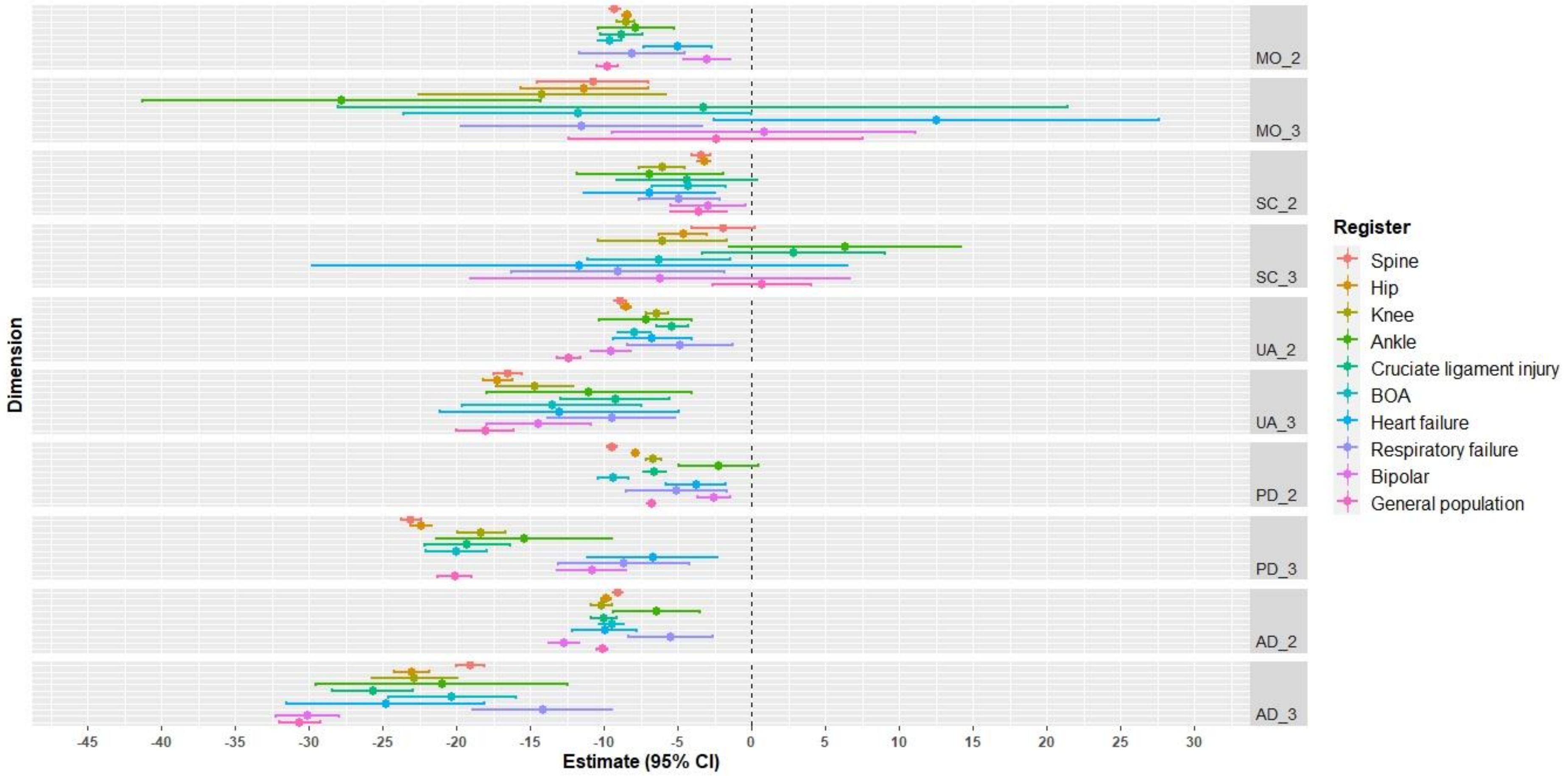

Figure S5: Estimates, OLS model, 1-year

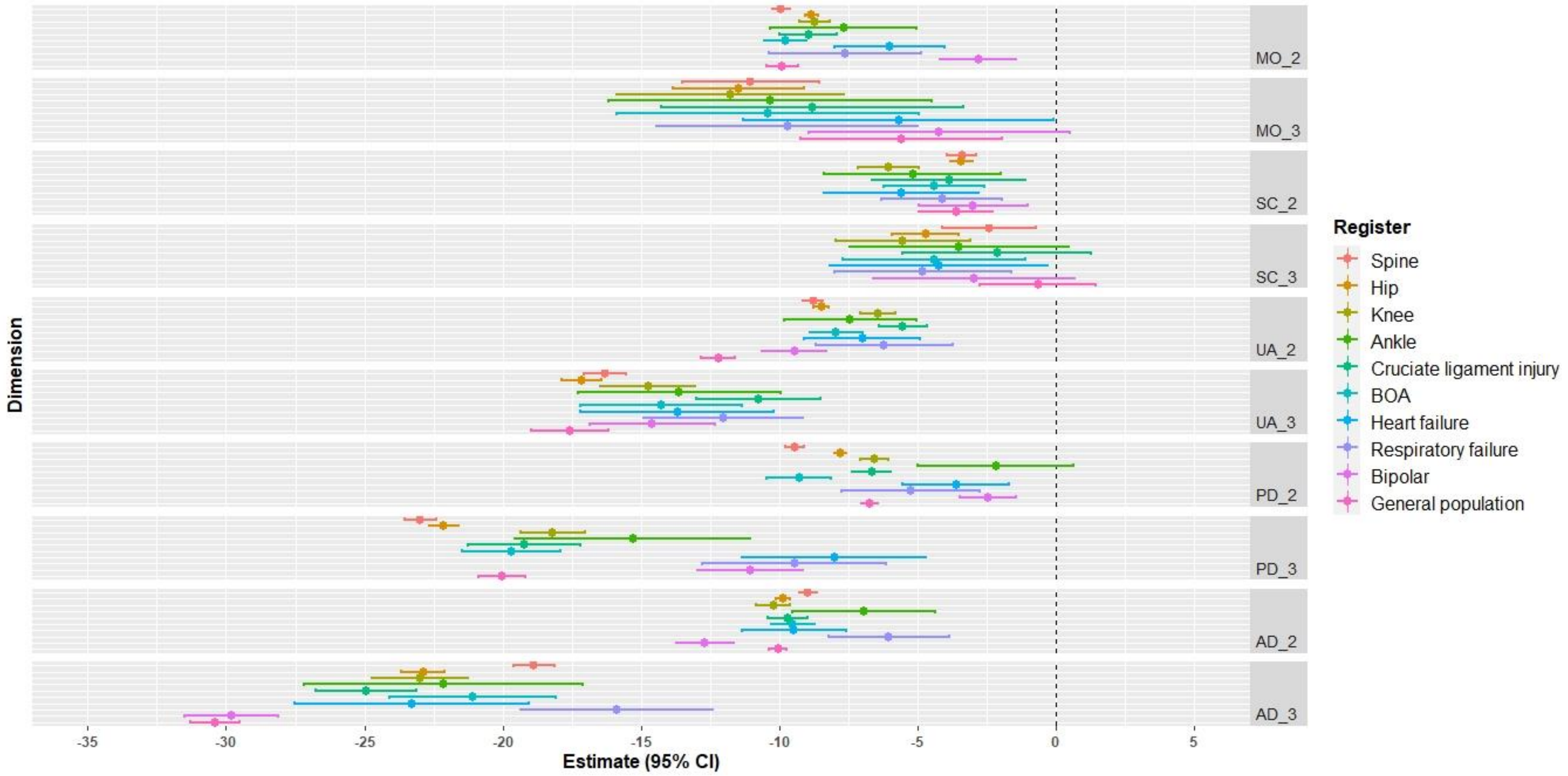

Figure S6: Estimates, mixed model, 1-year

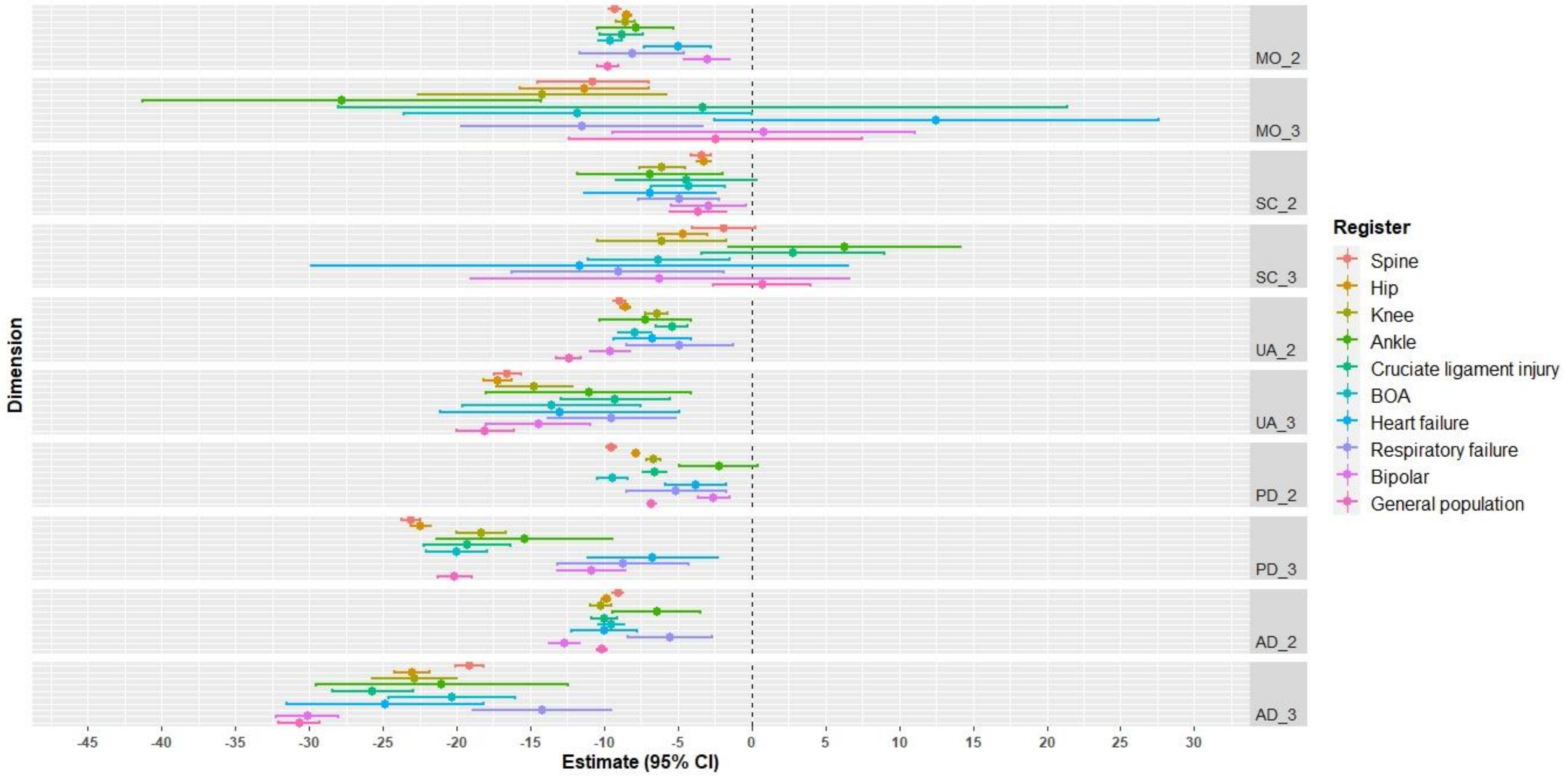

Figure S7: Estimates, OLS model, 1-year, adjusted for sex and age groups

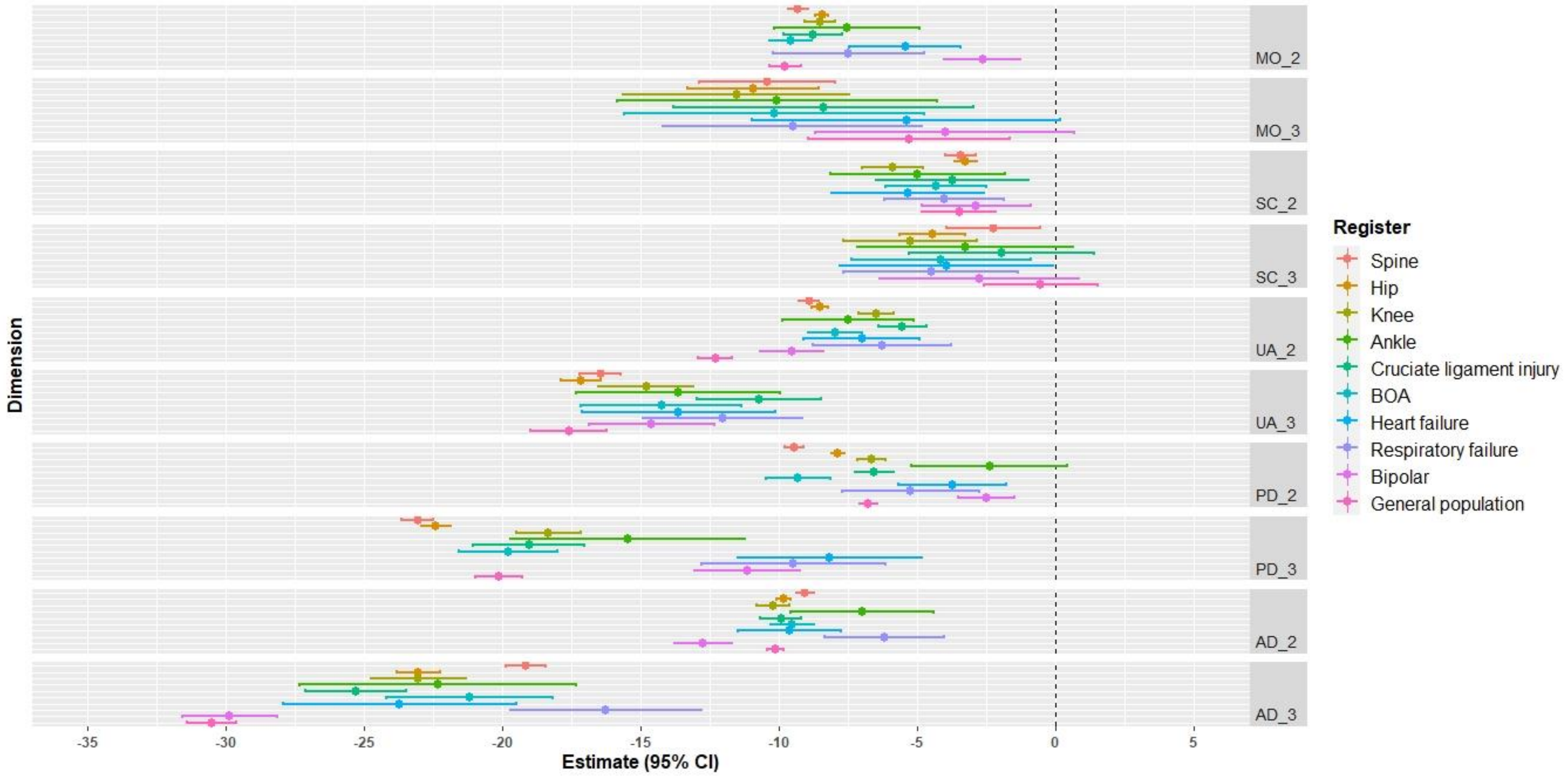

Figure S8: Estimates, mixed model, 1-year, adjusted for sex and age groups

Table S9: Ordinary least squares regressions in the pooled data at baseline

Parameters	Model 1<br>Pooled (patient data only)			Model 2<br>Pooled data (including general population)			Model 3<br>Pooled data (adjusted for patient group)		
	**Estimate**	**RSE**	**P-value**	**Estimate**	**RSE**	**P-value**	**Estimate**	**RSE**	**P-value**
Intercept	77.42	0.25	<.0001	86.30	0.08	<.0001	87.47	0.08	<.0001
MO_2	-5.45	0.13	<.0001	-8.08	0.11	<.0001	-6.90	0.13	<.0001
MO_3	-12.52	0.67	<.0001	-14.39	0.66	<.0001	-11.85	0.66	<.0001
SC_2	-3.84	0.14	<.0001	-3.60	0.14	<.0001	-3.52	0.14	<.0001
SC_3	-2.11	0.54	<.0001	-1.85	0.52	0.0003	-2.20	0.51	<.0001
UA_2	-6.81	0.11	<.0001	-7.70	0.10	<.0001	-6.47	0.10	<.0001
UA_3	-13.72	0.19	<.0001	-14.38	0.19	<.0001	-12.43	0.19	<.0001
PD_2	-3.57	0.26	<.0001	-9.22	0.12	<.0001	-7.27	0.13	<.0001
PD_3	-13.14	0.28	<.0001	-18.81	0.10	<.0001	-16.56	0.17	<.0001
AD_2	-8.71	0.10	<.0001	-8.94	0.09	<.0001	-8.49	0.09	<.0001
AD_3	-18.07	0.26	<.0001	-20.08	0.25	<.0001	-19.66	0.25	<.0001
Spine							-9.78	0.17	<.0001
Hip							-4.92	0.16	<.0001
Knee							0.37	0.21	0.0795
Ankle							-3.88	0.74	<.0001
Cruciate ligament							-7.19	0.25	<.0001
BOA							-2.42	0.23	<.0001
Heart failure							-9.13	0.52	<.0001
Respiratory failure							-11.41	0.77	<.0001
Bipolar							-7.31	0.29	<.0001
Adjusted R^2	0.2691			0.3958			0.4125		
RMSE	19.48			18.57			18.31		

BOA: Better management of OsteoArthritis; reference group in Model 3: general population; RMSE: Root mean square error; RSE: Robust standard error; Darker shades under estimate columns show inconsistency in decrement; Lighter shades in the P-value columns show non-statistically significant estimates

Table S10: Ordinary least squares regressions in the pooled data at 1-year follow-up

Parameters	Model 1<br>Pooled (patient data only)			Model 2<br>Pooled data (including general population)			Model 3<br>Pooled data (adjusted for patient group)		
	Estimate	RSE	P-value	Estimate	RSE	P-value	Estimate	RSE	P-value
Intercept	88.80	0.05	<.0001	88.81	0.04	<.0001	88.63	0.07	<.0001
MO_2	-8.58	0.09	<.0001	-8.73	0.09	<.0001	-9.14	0.09	<.0001
MO_3	-10.56	1.23	<.0001	-9.56	1.23	<.0001	-9.56	1.24	<.0001
SC_2	-3.43	0.18	<.0001	-3.26	0.18	<.0001	-3.45	0.18	<.0001
SC_3	-4.07	0.61	<.0001	-3.42	0.58	0.0003	-3.56	0.58	<.0001
UA_2	-8.50	0.11	<.0001	-8.90	0.11	<.0001	-8.72	0.11	<.0001
UA_3	-16.52	0.31	<.0001	-16.65	0.30	<.0001	-16.25	0.30	<.0001
PD_2	-8.09	0.08	<.0001	-7.82	0.07	<.0001	-7.53	0.07	<.0001
PD_3	-21.69	0.21	<.0001	-21.41	0.20	<.0001	-20.83	0.30	<.0001
AD_2	-10.27	0.10	<.0001	-10.19	0.09	<.0001	-9.81	0.09	<.0001
AD_3	-22.60	0.33	<.0001	-24.36	0.30	<.0001	-23.74	0.30	<.0001
Spine							-1.38	0.10	<.0001
Hip							0.92	0.08	<.0001
Knee							1.09	0.13	<.0001
Ankle							1.30	0.57	0.0220
Cruciate ligament							-1.72	0.19	<.0001
BOA							-1.21	0.19	<.0001
Heart failure							-5.72	0.50	<.0001
Respiratory failure							-5.61	0.69	<.0001
Bipolar							-4.54	0.27	<.0001
Adjusted R^2	0.5365			0.5330			0.5366		
RMSE	14.31			14.10			14.05		

BOA: Better management of OsteoArthritis; reference group in Model 3: general population; RMSE: Root mean square error; RSE: Robust standard error; Darker shades under estimate columns show inconsistency in decrement; Lighter shades in the P-value columns show non-statistically significant estimates

Table S11: Ordinary least squares models of EQ-5D-5L dimensions on EQ VAS score in the BOA and Hip registers

Variable	Register							
	BOA				Hip			
	Baseline		1-year follow-up		Baseline		1-year follow-up	
	Estimate	p-value	Estimate	p-value	Estimate	p-value	Estimate	p-value
Intercept	82.97	<0.001	89.14	<0.001	78.06	<0.001	88.76	<0.001
MO_2	-2.61	<0.001	-3.68	<0.001	-1.60	0.089	-2.77	<0.001
MO_3	-5.13	<0.001	-5.99	<0.001	-3.57	<0.001	-5.94	<0.001
MO_4	-7.52	<0.001	-9.48	<0.001	-5.34	<0.001	-9.90	<0.001
MO_5	-10.84	<0.001	-12.31	<0.001	-7.50	<0.001	-13.15	<0.001
SC_2	-2.58	<0.001	-2.75	<0.001	-2.14	<0.001	-2.04	<0.001
SC_3	-4.44	<0.001	-5.44	<0.001	-3.82	<0.001	-3.10	<0.001
SC_4	-7.24	<0.001	-6.62	<0.001	-8.09	<0.001	-4.72	<0.001
SC_5	1.66	0.451	8.07	0.173	-7.10	0.002	-0.14	0.971
UA_2	-3.06	<0.001	-3.33	<0.001	-2.21	0.001	-3.81	<0.001
UA_3	-6.01	<0.001	-6.52	<0.001	-4.51	<0.0001	-8.78	<0.001
UA_4	-9.00	<0.001	-9.04	<0.001	-7.98	<0.001	-12.51	<0.001
UA_5	-12.49	<0.001	-12.03	<0.001	-11.25	<0.001	-13.37	<0.001
PD_2	-0.71	0.230	-5.17	<0.001	3.26	0.294	-2.64	<0.001
PD_3	-5.28	<0.001	-11.24	<0.001	0.02	0.996	-7.92	<0.001
PD_4	-9.58	<0.001	-15.72	<0.001	-5.66	0.062	-13.82	<0.001
PD_5	-16.65	<0.001	-25.15	<0.001	-13.35	<0.001	-15.27	<0.001
AD_2	-5.75	<0.001	-5.85	<0.001	-5.92	<0.001	-6.38	<0.001
AD_3	-11.72	<0.001	-12.16	<0.001	-11.60	<0.001	-12.64	<0.001
AD_4	-17.78	<0.001	-18.16	<0.001	-16.87	<0.001	-19.42	<0.001
AD_5	-23.30	<0.001	-26.39	<0.001	-17.73	<0.001	-29.42	<0.001
**Adjusted R2**	0.3287		0.4530		0.2524		0.5572	
**n**	53,330		12,520		20,922		8,395	

BOA: Better management of OsteoArthritis; reference group in Model 3: general population; RMSE: Root mean square error; RSE: Robust standard error; Darker shades under estimate columns show inconsistency in decrement; Lighter shades in the P-value columns show non-statistically significant estimates
